# Changes of DNA methylation in smokers and ex-smokers referred for coronary angiography. Results from the LURIC study

**DOI:** 10.1186/s13148-026-02118-9

**Published:** 2026-04-04

**Authors:** Graciela E. Delgado, Angela P. Moissl-Blanke, Bernhard K. Krämer, Stefan Lorkowski, Rüdiger Siekmeier, Daniel Duerschmied, Norbert Frey, Winfried März, Marcus E. Kleber

**Affiliations:** 1 LURIC Study GmbH, Josef-Mörtl-Straße 23, Aystetten, Germany; 2 Department of Medicine I (Cardiology, Hemostaseology, Medical Intensive Care), Medical Faculty Mannheim, Heidelberg University, Mannheim, Germany; 3 Medical Faculty Mannheim, Heidelberg University, Mannheim, Germany; 4 Institute of Nutritional Sciences, Friedrich Schiller University Jena, Jena, Germany; 5 Competence Cluster for Nutrition and Cardiovascular Health (nutriCARD) Halle-Jena-Leipzig, Jena, Germany; 6 Federal Institute for Drugs and Medical Services, Bonn, Germany; 7 European Center for AngioScience (ECAS), German Centre for Cardiovascular Research (DZHK) Partner Site Heidelberg/Mannheim, and Centre for Cardiovascular Acute Medicine Mannheim (ZKAM), Medical Centre Mannheim and Medical Faculty Mannheim, Heidelberg University, Mannheim, Germany; 8 Department of Medicine III (Cardiology, Angiology and Pneumology), University Hospital Heidelberg, Heidelberg, Germany; 9 Clinical Institute of Medical and Chemical Laboratory Diagnostics, Medical University Graz, Graz, Austria; 10 SYNLAB Academy, SYNLAB Holding Deutschland GmbH, Augsburg and Mannheim, Germany; 11 SYNLAB MVZ Humangenetik Mannheim, Mannheim, Baden-Württemberg Germany

**Keywords:** Epigenetics, Smoking, Mortality, DNA methylation

## Abstract

**Background:**

Tobacco smoking remains a major global health burden and is a leading risk factor for cardiovascular disease and cancer. Accumulating evidence suggests that tobacco smoke induces widespread alterations in DNA methylation, which may contribute to smoking-related morbidity and mortality.

**Methods:**

The Ludwigshafen Risk and Cardiovascular Health (LURIC) study is a monocentric prospective cohort including 3316 patients referred for coronary angiography. Genome-wide DNA methylation was assessed in 2423 participants using the Illumina HumanMethylationEPIC BeadChip. A discovery–replication design was applied (discovery *n* = 1262; replication *n* = 1161). Associations between smoking status (never/former vs. current) and CpG-specific methylation levels were evaluated using multivariable linear regression models. Cox proportional hazards models were used to assess associations with all-cause and cardiovascular mortality. Mediation was examined within a counterfactual framework using natural effect models.

**Results:**

In the discovery sample, 14,403 CpG sites were significantly associated with smoking after false-discovery-rate correction (differentially methylated probes, DMPs). Of these, 3,000 were replicated in the independent sample at an FDR-adjusted p value < 0.05. In random-effect meta-analysis of both samples, 24,930 DMPs remained significant after multiple testing correction, of which 11,907 had not been reported in the largest published smoking EWAS to date. Among former smokers, a subset of DMPs remained differentially methylated more than 10 years after smoking cessation, indicating long-term persistence of smoking-associated epigenetic alterations. A CpG score constructed from mortality-associated DMPs was strongly associated with all-cause mortality. Inclusion of this score in Cox regression models attenuated the association between smoking status and mortality. Mediation analysis demonstrated a statistically significant natural indirect effect of smoking on all-cause mortality via the CpG score.

**Conclusions:**

Tobacco smoking is associated with widespread, exposure-dependent alterations in DNA methylation, many of which persist for years after cessation. These epigenetic changes are strongly linked to mortality risk and may represent an important biological pathway underlying the association between smoking and adverse health outcomes.

**Supplementary Information:**

The online version contains supplementary material available at 10.1186/s13148-026-02118-9.

## Background

Tobacco smoking remains a major public health issue worldwide, contributing to a broad spectrum of diseases, including cardiovascular diseases, cancer, and chronic obstructive pulmonary disease [1]. Although the harmful health effects of smoking are well-established, the underlying molecular mechanisms driving these effects are still being intensively investigated [2]. Advances in epigenetics, particularly in understanding DNA methylation and its role in gene regulation, have revealed a novel layer of complexity in how tobacco smoke influences gene expression without altering the underlying DNA sequence. Methylation at CpG sites can regulate gene expression by modulating transcriptional activity, often leading to gene silencing when methylation occurs in promoter regions [3].Tobacco smoke, which contains thousands of chemicals, including known carcinogens and mutagens, can significantly alter methylation patterns across the genome [4]. In recent years, epigenome-wide association studies (EWAS) have shown impacts of smoking on the methylation pattern [5]. A number of smoking-associated blood DNA methylation biomarkers have been identified. Among these markers, CpGs at the loci AHRR (aryl-hydrocarbon-receptor-repressor), F2RL3 (F2R-like thrombin/trypsin receptor 3), 2q37.1 (ALPPL2, alkaline phosphatase, placental-like 2) and 6p21.33 (IER, immediate early response 3) were common among most differentially methylated sites [6, 8].

Some of these smoking-induced epigenetic changes are reversible upon smoking cessation. Evidence suggests that within five years of quitting smoking, the methylation levels of many CpG sites begin to return to levels similar to those observed in never-smokers [8, 9]. Certain genes, however, particularly some involved in critical regulatory pathways, remain epigenetically altered for decades after cessation. For example, persistent hypermethylation of the F2RL3 gene, which encodes a protein involved in coagulation and inflammatory response, has been observed even 30 years post-cessation [6, 10]. So, some of the smoking-induced changes in DNA methylation can lead to a “epigenetic memory”, which means that even former smokers have an increased risk of diseases such as cancer, because some of the epigenetic changes persist.

In our study, we analyzed the association of smoking with DNA methylation in current smokers, ex-smokers and never smokers from the Ludwigshafen Risk and Cardiovascular Health (LURIC) study, a monocentric prospective study that recruited patients referred for coronary angiography. Furthermore, the retrospective analysis of former smokers allowed us the identification of CpG sites that remain differentially methylated for more than 10 years after smoking cessation.

## Materials and methods

### Study subjects

The Ludwigshafen Risk and Cardiovascular Health (LURIC) study included 3316 individuals who had been hospitalized for coronary angiography at the Klinikum Ludwigshafen, a tertiary care center in Southwestern Germany [11]. Clinical indications for angiography were chest pain or a positive non-invasive stress test suggestive of myocardial ischemia. Individuals suffering from acute illnesses other than acute coronary syndrome, chronic non-cardiac diseases and a history of malignancy within the past five years were excluded. The study was approved by the ethics committee at the “State Chamber of Physicians of Rhineland-Palatinate” (“Landesärztekammer Rheinland Pfalz”). All patients signed informed written consent before study start. The presence of a visible luminal narrowing (> 20% stenosis) in at least one of 15 coronary segments was used to define coronary artery disease (CAD) according to the classification of the American Heart Association [11]. Diabetes mellitus (DM) was defined according to 2010 guidelines of the American Diabetes Association as increased fasting (≥ 126 mg/dl) and/or post-challenge (2 h after the 75 g glucose load > 200 mg/dl) glucose and/or elevated glycated hemoglobin (≥ 6.5%) and/or history of DM. Hypertension was defined as a systolic and/or diastolic blood pressure ≥ 140 and/or ≥ 90 mm Hg or a history of hypertension. Smoking status was assessed based on a questionnaire and verified by measurement of serum cotinine concentration. A cut-off of 15 µg/l was used to reclassify self-reported non- or ex-smokers as active smokers. The group of former smokers was subdivided into those that had quit smoking less than 10 years before study entry and those who had quit more than 10 years before.

### Laboratory procedures

Fasting blood samples were obtained by venipuncture in the early morning. A detailed summary of analytic methods has been reported previously [11]. The lipoproteins were separated using a combined ultracentrifugation-precipitation method (β-quantification). Cholesterol was measured with enzymatic reagents from WAKO on a WAKO 30 R or Olympus AU640 analyser. Triglycerides were quantified with an enzymatic assay on a Hitachi 717 analyser (Roche). High-sensitive C-reactive protein (hsCRP), serum amyloid A (SAA) and cystatin C were measured by immunonephelometry (N-High-Sensitive CRP; N LATEX SAA; N-Latex Cystatin C, Dade Behring, Marburg, Germany) using a Behring nephelometer II.

### Infinium HumanMethylation EPIC Beadchip

DNA methylation was assessed using the Illumina HumanMethylation EPIC Beadchip (Illumina). The analyses were performed at Life&Brain (Bonn, Germany). Due to budget contrains as well as the availability of a sufficient amount of DNA the analyses were done in two batches. The first batch was measured in 2017 and was used as the discovery sample in the current analysis. The second batch was measured in 2018 and was used as replication sample. The quality control of the methylation data was done using the well-defined CPACOR pipeline [12], excluding samples with a call rate ≤ 95% and those that showed sex discordance. We also applied a CpGs quality control and removed those CpGs located in close proximity (1–2 bp) of a genetic polymorphism in the European population with a frequency > 0.01 as well as cross-reactive probes. For the discovery sample 1262 patients remained for analyses and for the replication sample 1161 samples were available after quality control.

We also removed CpGs with a detection p-value > 0.05 in at least 1% of the samples and performed quantile normalization. Cell counts of leukocytes were estimated using Houseman’s algorithm [13] as implemented in the ‘minfi’ R package version 1.18.4 [14]. The distribution of calculated cell types in discovery and replication sample is shown in Supplementary Figs. 1–14. To adjust for batch and technical effects we performed a principal component analysis (PCA) of control sample intensities, and then included the first 30 principal components (PCs) as linear predictors in the regression analysis. After the quality control, β-values [ranging between 0 (no methylation) and 1 (full methylation)] were calculated according to the following equation:

β = M/(M + U + 100), where M and U denote the methylated and the unmethylated signal, respectively.

### Statistical analysis

Continuous variables were compared between groups using ANOVA with non-normally distributed variables being logarithmically transformed before entering analyses. Categorical variables were compared between groups using the χ^2^ test.

DNA-methylation analyses were conducted using the R package “CpGassoc” version 2.70 [15]. All the analyses were performed using multivariable linear regression. In each model the methylation β-value was considered as the dependent variable and smoking status as an independent variable, with adjustment for sex, age, estimated cell count, the first 30 PCs of control probe intensities and the array ID. Smoking status was treated as a categorical variable.

The gene annotation for each probe was based on the manufacturer’s annotation file. We performed EWAS of smoking status and used the Bonferroni-Holm procedure and the false-discovery-rate procedure after Benjamini-Hochberg to correct for multiple testing separately in the discovery sample and the replication sample and also performed a meta-analysis of both datasets using the R package “meta” v 8.2-1.2 [16]. To calculate the inflation and bias of test statistics we used the R package “bacon” v 1.38.0 [17].

For the comparison of our results with published data we downloaded data recently published by Hoang et al. [18].

Gene set enrichment analysis of the differentially methylated CpG sites was performed using the gometh function from the R package “missMethyl” v 1.44.0 [19]. Kyoto Encyclopedia of Genes and Genomes (KEGG) pathway analyses and Gene ontology (GO) enrichment were conducted for the differentially methylated CpG sites obtained in the meta-analysis of current smokers vs. never smokers as well as for the subgroup of DMPs that were not reported by Hoang et al.

To create a weighted score based on 5 CpGs that were significantly associated with mortality, we multiplied each individual beta value with the effect size from the meta-analysis and summed these values up. Before entering Cox regression analyses the CpG score was Z-transformed.

Cox proportional hazard models were built to assess the association with all-cause mortality and cardiovascular mortality. The proportional hazards assumption was assessed using Schoenfeld residuals and formal tests based on scaled Schoenfeld residuals (cox.zph function in R). No meaningful violations were observed.

DNA methylation was evaluated as a potential mediator of the association between smoking status and all-cause mortality using a counterfactual mediation framework. The mediator model was specified as a linear regression of methylation on smoking status and covariates, and the outcome model as a Cox proportional hazards model including an exposure–mediator interaction term. Analyses were performed using the R package “regmedint’” version 1.0.1, [20]The R package “pROC” 1.19.0.1 [21] was used to analyze ROC Curves. Venn diagrams were plotted using the R package “ggVennDiagram” 1.5.7 [22].

All statistical analysis was performed using SPSS 27.0 (IBM SPSS, Chicago, IL, USA) and R version 4.5.2 (http://www.r-project.org) [23].

## Results

### Characteristics of the study population

We conducted a genome-wide analysis of DNA methylation using the Illumina Human Methylation EPIC Bead Chip on peripheral blood DNA from participants of the LURIC study. A total of 1262 individuals in the discovery cohort and 1161 in the replication cohort were included following stringent quality control procedures. Participants were stratified into current, former, and never smokers. The characteristics of these groups, including key metabolic and cardiovascular risk factors, are summarised in Table 1. No preselection was performed when choosing the samples and both groups differed in some baseline characteristics. A comparison of both groups is presented in Supplementary Table 1. The most significant differences between both groups are higher percentages of men and participants with CAD in the replication sample, as well as lower low density lipoprotein cholesterol (LDL-C) concentration and lower estimated glomerular filtration rate (eGFR).

**Table 1:** Study characteristics of discovery samples and replications samples

*N*	Discovery sample (*n* = 1262)				Replication sample (*n* = 1161)			
	Never smoker	Former smoker	Current smoker	*P*	Never smoker	Former smoker	Current smoker	*P*
	423	539	300		459	453	249	
Age (years)	64.9(10.6)	63.9(9.84)	55.8(10.1)	< 0.001	65.9(9.77)	64.6(9.24)	56.7(10.8)	< 0.001
Female sex (%)	52.0	12.1	23.7	< 0.001	59.5	14.6	22.9	< 0.001
BMI (kg/m^2^)	27.2(4.32)	27.7(3.85)	27.1(4.39)	0.064	27.6(4.14)	27.8(3.81)	27.1(4.27)	0.098
LDL-C (mg/dl)	123(34.0)	115(30.9)	121(31.4)	< 0.001	113(36.4)	106(34)	110(32.6)	0.008
HDL-C (mg/dl)	41.1(10.6)	37.7(10.8)	35.2(9.72)	< 0.001	41(11.6)	38.5(10.1)	36.8(10.8)	< 0.001
TG (mg/dl)	136(105–190)	156(114–210)	156(115–228)	< 0.001	133(101–190)	147(112–194)	162(114–217)	0.001
Systolic BP (mmHg)	145(23.6)	143(22.9)	134(22.9)	< 0.001	143(23.3)	143(23.5)	134(22.8)	< 0.001
Diastolic BP (mmHg)	82.0(11.5)	81.9(11.2)	78.7(11.7)	< 0.001	80.9(11.0)	81.7(11.8)	79.8(11.4)	0.108
eGFR (ml/min/1.73m^2^)	81.7(18.5)	81.6(20.0)	88.5(19.4)	< 0.001	76.3(19.5)	79.9(20.4)	87.7(20.2)	< 0.001
hsCRP (mg/l)	2.44(1.10–6.60)	3.66(1.30–8.93)	5.24(2.11–10.2)	< 0.001	2.75(1.29–7.29)	3.18(1.24–9.04)	4.46(1.43–9.80)	0.011
Leucocytes (/nl)	6.3(5.38–7.50)	6.6(5.6–8.6)	7.72(6.46–9.20)	< 0.001	6.42(5.60–7.60)	6.70(5.70–7.98)	7.98(6.60–9.62)	< 0.001
Alcohol (g eth/d)	0.963(0–12)	6(0–24.1)	2.94(0–21.1.1)	< 0.001	1.68(0–24)	20(0–30)	4.48(0–44)	< 0.001
CD8T cells (%)	6.41(3.06–9.32)	5.7(3.02–8.79)	6.28(2.85–8.8)	0.053	6.67(3.73–9.39)	5.56(2.86–8.61)	5.69(3.26–8.33)	0.388
CD4T cells (%)	12.7(8.48–17.2)	12.4(8.54–17.5)	13.9(9.17–18.7)	0.503	12.3(8.77–16.7)	12.3(8.47–16.1)	14.2(10.5–18.5)	0.002
NK cells (%)	5.51(2.91–8.79)	5.07(2.00–8.21)	2.19(0–5.83)	< 0.001	5.84(2.88–9.32)	5.59(2.96–9.73)	3.01(0.414–5.82)	< 0.001
B cell (%)	3.40(1.50–6.11)	3.59(1.39–5.57)	3.34(0.96–5.67)	0.069	4.05(2.67–5.47)	3.97(2.59–5.38)	4.34(2.72–5.74)	0.604
Monocytes (%)	7.39(5.86–9.26)	8.44(6.83–10.4)	7.44(5.55–9.27)	< 0.001	7.25(5.63–8.78)	7.28(5.71–8.91)	6.86(5.34–8.76)	0.700
Eosinophiles (%)	0(0–0)	0(0–0)	0(0–0)	0.311	0(0–0)	0(0–0)	0(0–0)	0.927
Neutrophiles (%)	62.7(55.3–70.6)	63.6(56.1–70.9)	64.6(57.2–73.4)	0.004	63.5(57.2–69.4)	64.6(57.5–70.0)	65.2(58.9–70.5)	0.317
Coronary artery disease (%)	72.8	90.0	79.0	< 0.001	64.7	80.6	79.5	< 0.001
Diabetes mellitus (%)	35.0	39.0	35.3	0.376	40.7	45.7	38.6	0.134
Pack years	-	21(10–40)	27.5(15–40)	-	-	20(8–40)	30(15–45.5)	-

Data given as mean values (standard deviation) or median values (25th −75th percentile) or percentages for categorical variables. Differences between smoking groups were assessed using ANOVA or chi-square test. BMI: body mass index, LDL-C: low density lipoprotein-cholesterol, TG: triglycerides, eGFR=estimated glomerular filtration rate, hsCRP= high-sensitive C reactive protein

### Differentially methylated probes (DMP) in response to smoking

**Fig. 1:**
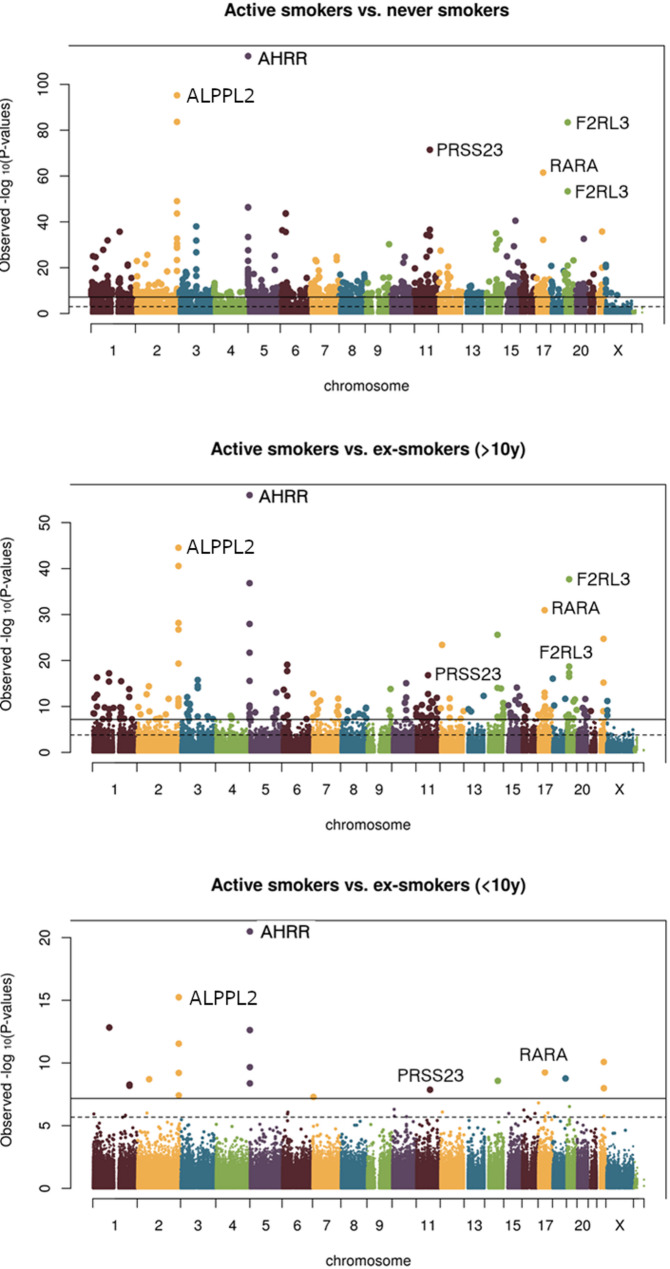
Manhattan plots of EWAS between active smokers, never smokers and ex-smokers. The methylation sites with the lowest overall P values are labeled with the name of the adjacent gene. The solid line indicates the significance threshold after FDR correction for multiple testing, and the dashed line indicates the significance threshold after Bonferroni-Holm correction

The results of the analysis of current vs. never smokers in both the discovery and the replication sample are shown as Manhattan plot in Fig. 1A and QQ plots are presented in Supplementary Fig. 15. In the discovery sample we detected 1071 CpGs that had significantly different methylation levels after conservative Bonferroni-Holm correction and 14,403 CpGs that had significantly different methylation levels after FDR correction. Of these 14,403 CpGs 3,000 were replicated in the replication sample at an FDR-adjusted P value of < 0.05 (Supplementary Table 2). In Supplementary Fig. 16 the effect sizes in discovery and replication samples are shown as a scatter plot. The Person correlation coefficient was 0.417. Supplementary Fig. 17 shows reflective Manhattan plots of the analyses with smoking status used as numerical variable with CpGs with hypermethylation depicted in the upper half and CpGs with hypomethylation in the lower half. We calculated the inflation and the bias of test statistics using the “bacon” package in R. For the EWAS of current smokers vs. never smokers, the discovery sample inflation was estimated to be 1.17 and bias was estimated to be 0.03. The corresponding values for the replication sample were 1.11 for the inflation and 0.06 for the bias.

In Table 2, the 20 most significant CpGs from the discovery sample are shown together with the median (25th to 75th percentile) methylation beta value in both samples. Full lists of all significantly different CpGs for the discovery sample and the replication sample are given in Supplementary Tables 3 and 4, respectively Tables 3 and 4.

**Table 2:** Top differently methylated CpGs active smoker vs. never-smokers

CpG	CHR	Position	Gene	Discovery sample				Replication sample			
				Effect size	β smoker	β nonsmoker	FDR	Effect size	β smoker	β nonsmoker	FDR
cg05575921	5	373,378	AHRR	−0.257	0.566(0.508–0.661)	0.878(0.856–0.895)	3.35E-107	−0.234	0.567(0.509–0.649)	0.860(0.836–0.875)	5.78E-94
cg21566642	2	233,284,661	ALPPL2	−0.159	0.380(0.348–0.434)	0.557(0.519–0.591)	2.21E-90	−0.143	0.387(0.351–0.430)	0.550(0.510–0.584)	2.09E-72
cg01940273	2	233,284,934	ALPPL2	−0.110	0.458(0.433–0.49)	0.575(0.545–0.608)	5.62E-79	−0.096	0.447(0.418–0.472)	0.553(0.527–0.583)	5.39E-64
cg03636183	19	17,000,585	F2RL3	−0.132	0.500(0.462–0.552)	0.646(0.618–0.677)	6.96E-79	−0.111	0.480(0.443–0.534)	0.609(0.587–0.631)	7.97E-62
cg14391737	11	86,513,429	PRSS23	−0.124	0.254(0.211–0.312)	0.400(0.351–0.448)	5.06E-67	−0.107	0.243(0.204–0.298)	0.374(0.338–0.408)	2.01E-42
cg17739917	17	38,477,572	RARA	−0.091	0.308(0.269–0.352)	0.401(0.367–0.441)	3.89E-57	−0.090	0.276(0.241–0.314)	0.375(0.342–0.414)	9.16E-44
cg21911711	19	16,998,668	F2RL3	−0.073	0.698(0.669–0.74)	0.78(0.753–0.811)	4.98E-49	−0.072	0.676(0.641–0.718)	0.755(0.728–0.778)	9.59E-45
cg17087741	2	233,283,010	ALPPL2	−0.077	0.824(0.777–0.867)	0.900(0.885–0.914)	8.34E-45	−0.078	0.807(0.751–0.84)	0.881(0.865–0.897)	1.48E-41
cg26703534	5	377,358	AHRR	−0.054	0.626(0.596–0.661)	0.694(0.669–0.718)	3.19E-42	−0.056	0.601(0.568–0.627)	0.666(0.647–0.682)	1.08E-43
cg25648203	5	395,444	AHRR	−0.065	0.721(0.679–0.76)	0.792(0.766–0.811)	3.98E-42	−0.062	0.696(0.654–0.73)	0.763(0.742–0.778)	4.42E-38
cg15342087	6	30,720,209	IER3	−0.041	0.817(0.792–0.842)	0.859(0.845–0.871)	1.24E-39	−0.035	0.786(0.756–0.811)	0.824(0.812–0.838)	6.83E-25
cg03329539	2	233,283,329	ALPPL2	−0.055	0.325(0.301–0.363)	0.387(0.360–0.416)	1.40E-39	−0.057	0.307(0.279–0.333)	0.368(0.341–0.396)	3.82E-31
cg24859433	6	30,720,203	IER3	−0.046	0.802(0.78–0.831)	0.851(0.835–0.866)	1.54E-39	−0.043	0.782(0.752–0.803)	0.824(0.810–0.837)	2.59E-34
cg18110140	15	75,350,380		−0.084	0.405(0.358–0.464)	0.519(0.483–0.558)	1.69E-36	−0.087	0.383(0.336–0.429)	0.498(0.458–0.531)	4.91E-34
cg19859270	3	98,251,294	GPR15	−0.029	0.880(0.86–0.892)	0.907(0.897–0.916)	4.82E-34	−0.026	0.859(0.841–0.877)	0.885(0.876–0.897)	1.45E-20
cg14466441	6	11,392,193		−0.048	0.806(0.783–0.838)	0.853(0.833–0.874)	1.18E-32	−0.049	0.776(0.751–0.805)	0.821(0.792–0.845)	3.62E-35
cg00475490	11	86,517,110	PRSS23	−0.052	0.091(0.0769–0.113)	0.134(0.111–0.167)	2.00E-32	−0.052	0.0842(0.071–0.105)	0.130(0.107–0.153)	3.13E-20
cg05086879	22	39,861,490	MGAT3	−0.052	0.763(0.725–0.797)	0.812(0.788–0.832)	6.97E-32	−0.051	0.736(0.700–0.772)	0.792(0.773–0.81)	2.09E-28
cg00045592	1	160,714,299	SLAMF7	−0.070	0.434(0.373–0.485)	0.511(0.462–0.556)	7.64E-32	−0.071	0.404(0.365–0.448)	0.484(0.451–0.514)	4.07E-29
cg14753356	6	30,720,108	IER3	−0.053	0.366(0.308–0.417)	0.417(0.366–0.472)	1.03E-31	−0.050	0.336(0.294–0.38)	0.384(0.347–0.426)	7.23E-23

**Table 3:** Top differently methylated CpGs active smoker vs. ex-smokers (≥ 10 years)

CPG.Labels				Discovery sample			Replication sample		
	CHR	Position	Gene	Beta smoker	β ex-smoker ≥ 10 years	FDR	Beta smoker	β ex-smoker ≥ 10 years	FDR
cg05575921	5	373,378	AHRR	0.566(0.508–0.661)	0.818(0.752–0.867)	7.70E-51	0.567(0.509–0.649)	0.808(0.742–0.849)	1.79E-40
cg01940273	2	233,284,934	ALPPL2	0.458(0.433–0.49)	0.537(0.512–0.572)	1.08E-39	0.447(0.418–0.472)	0.518(0.485–0.551)	8.18E-29
cg21566642	2	233,284,661	ALPPL2	0.380(0.348–0.434)	0.496(0.459–0.541)	6.80E-36	0.387(0.351–0.43)	0.497(0.453–0.535)	1.49E-31
cg03636183	19	17,000,585	F2RL3	0.500(0.462–0.552)	0.613(0.575–0.643)	3.92E-33	0.48(0.443–0.534)	0.590(0.551–0.618)	3.31E-24
cg26703534	5	377,358	AHRR	0.626(0.596–0.661)	0.691(0.670–0.717)	2.24E-32	0.601(0.568–0.627)	0.666(0.647–0.685)	2.45E-24
cg17739917	17	38,477,572	RARA	0.308(0.269–0.352)	0.381(0.340–0.412)	1.43E-26	0.276(0.241–0.314)	0.346(0.314–0.381)	2.28E-22
cg03329539	2	233,283,329	ALPPL2	0.325(0.301–0.363)	0.373(0.344–0.401)	7.16E-24	0.307(0.279–0.333)	0.342(0.319–0.366)	1.13E-10
cg25648203	5	395,444	AHRR	0.721(0.679–0.760)	0.782(0.759–0.802)	1.03E-23	0.696(0.654–0.73)	0.759(0.735–0.776)	2.78E-19
cg17087741	2	233,283,010	ALPPL2	0.824(0.777–0.867)	0.887(0.861–0.904)	1.55E-22	0.807(0.751–0.84)	0.866(0.839–0.885)	6.43E-15
cg02738868	14	74,221,164	ELMSAN1	0.253(0.221–0.279)	0.273(0.248–0.293)	1.85E-21	0.247(0.227–0.264)	0.261(0.242–0.278)	6.40E-18
cg05086879	22	39,861,490	MGAT3	0.763(0.725–0.797)	0.817(0.799–0.835)	1.31E-20	0.736(0.700–0.772)	0.794(0.776–0.817)	8.58E-17
cg07986378	12	11,898,284	ETV6	0.546(0.487–0.597)	0.604(0.549–0.644)	2.48E-19	0.516(0.482–0.562)	0.555(0.514–0.591)	3.51E-4
cg04551776	5	393,366	AHRR	0.738(0.711–0.769)	0.781(0.758–0.804)	1.15E-17	0.715(0.686–0.741)	0.762(0.741–0.781)	2.03E-07
cg12956751	2	233,246,922	ALPP	0.596(0.565–0.623)	0.612(0.589–0.642)	2.49E-15	0.567(0.541–0.592)	0.587(0.562–0.609)	1.37E-10
cg15342087	6	30,720,209	IER3	0.817(0.792–0.842)	0.850(0.833–0.864)	4.07E-15	0.786(0.756–0.811)	0.813(0.795–0.83)	1.25E-07
cg21911711	19	16,998,668	F2RL3	0.698(0.669–0.740)	0.746(0.714–0.780)	9.03E-15	0.676(0.641–0.718)	0.726(0.693–0.755)	5.56E-12
cg24859433	6	30,720,203	IER3	0.802(0.780–0.831)	0.838(0.819–0.853)	8.86E-14	0.782(0.752–0.803)	0.814(0.792–0.833)	6.32E-13
cg10765427	19	17,005,225	CPAMD8	0.516(0.496–0.542)	0.549(0.530–0.568)	2.05E-13	0.494(0.476–0.516)	0.527(0.510–0.542)	2.85E-10
cg09935388	1	92,947,588	GFI1	0.621(0.543–0.695)	0.732(0.679–0.773)	2.34E-13	0.591(0.527–0.672)	0.717(0.660–0.752)	5.95E-14
cg21611682	11	68,138,269	LRP5	0.522(0.494–0.554)	0.559(0.538–0.582)	5.95E-13	0.498(0.473–0.518)	0.536(0.513–0.558)	1.47E-11

**Table 4:** Top differently methylated CpGs active smoker vs. ex-smokers (< 10 years)

CPG.Labels	CHR	Position	Gene	Discovery sample			Replication sample		
				Beta smoker	Beta ex-smoker < 10 years	FDR	Beta smoker	Beta ex-smoker < 10 years	FDR
cg26703534	5	377,358	AHRR	0.626(0.596–0.661)	0.690(0.667–0.708)	2.42E-15	0.601(0.568–0.627)	0.668(0.650–0.686)	4.29E-14
cg01940273	2	233,284,934	ALPPL2	0.458(0.433–0.490)	0.516(0.486–0.544)	2.12E-10	0.447(0.418–0.472)	0.508(0.482–0.534)	5.00E-12
cg05157376	1	92,781,750	RPAP2	0.599(0.535–0.643)	0.648(0.615–0.693)	3.65E-08	0.565(0.523–0.597)	0.603(0.558–0.651)	7.15E-06
cg05575921	5	373,378	AHRR	0.566(0.508–0.661)	0.730(0.68–0.802)	4.43E-08	0.567(0.509–0.649)	0.739(0.680–0.816)	1.12E-10
cg21566642	2	233,284,661	ALPPL2	0.380(0.348–0.434)	0.458(0.416–0.491)	4.27E-07	0.387(0.351–0.430)	0.468(0.427–0.523)	7.03E-12
cg05086879	22	39,861,490	MGAT3	0.763(0.725–0.797)	0.810(0.786–0.83)	1.02E-05	0.736(0.700–0.772)	0.785(0.766–0.811)	2.69E-06
cg04551776	5	393,366	AHRR	0.738(0.711–0.769)	0.775(0.752–0.799)	2.29E-05	0.715(0.686–0.741)	0.745(0.720–0.768)	0.54551191
cg03329539	2	233,283,329	ALPPL2	0.325(0.301–0.363)	0.362(0.335–0.388)	5.10E-05	0.307(0.279–0.333)	0.341(0.317–0.37)	0.0163501
cg17739917	17	38,477,572	RARA	0.308(0.269–0.352)	0.357(0.317–0.398)	5.10E-05	0.276(0.241–0.314)	0.329(0.300–0.369)	2.90E-06
cg07390844	18	72,935,911	TSHZ1	0.443(0.395–0.49)	0.493(0.447–0.537)	0.00012723	0.412(0.385–0.447)	0.461(0.426–0.486)	0.04716391
cg26673040	2	67,157,193	LOC101060019	0.780(0.755–0.805)	0.817(0.798–0.832)	0.00013324	0.759(0.735–0.784)	0.786(0.766–0.802)	0.01048778
cg02738868	14	74,221,164	ELMSAN1	0.253(0.221–0.279)	0.272(0.244–0.291)	0.00016381	0.247(0.227–0.264)	0.257(0.242–0.283)	6.17E-06
cg25648203	5	395,444	AHRR	0.721(0.679–0.760)	0.773(0.743–0.792)	0.00024174	0.696(0.654–0.73)	0.75(0.722–0.776)	6.52E-05
cg20295214	1	206,226,794	AVPR1B	0.768(0.743–0.792)	0.797(0.778–0.819)	0.00028454	0.741(0.717–0.762)	0.764(0.744–0.786)	0.06250958
cg08709672	1	206,224,334	AVPR1B	0.574(0.550–0.595)	0.600(0.579–0.619)	0.00032317	0.579(0.558–0.599)	0.603(0.586–0.626)	0.00587198
cg09338374	22	39,888,390		0.574(0.534–0.607)	0.542(0.524–0.576)	0.0004784	0.544(0.517–0.578)	0.520(0.487–0.559)	0.294754
cg19665316	11	77,590,553	INTS4	0.675(0.641–0.702)	0.669(0.632–0.692)	0.00059395	0.648(0.620–0.683)	0.640(0.604–0.674)	0.77312378
cg12956751	2	233,246,922	ALPP	0.596(0.565–0.623)	0.61(0.581–0.638)	0.00159389	0.567(0.541–0.592)	0.589(0.565–0.615)	0.04291682
cg09022230	7	5,457,225	TNRC18	0.649(0.614–0.681)	0.682(0.651–0.718)	0.00200819	0.612(0.582–0.648)	0.656(0.630–0.686)	0.00012067
cg05753553	17	2,689,486		0.723(0.692–0.754)	0.700(0.673–0.733)	0.00563237	0.701(0.669–0.726)	0.683(0.650–0.708)	0.45101231

In addition, a meta-analysis of both datasets was performed, and the corresponding P values are shown in Supplementary Table 5. For some CpGs we detected a heterogeneity of effect (e.g. for cg05575921 at the AHRR locus) and therefore we focus on the results of the random-effect analysis. In the random-effect meta-analysis there were 2763 CpGs with significantly different methylation levels after conservative Bonferroni-Holm correction and 24,930 were significant using the less conservative false-discovery-rate correction.

We compared our results with data recently published by Hoang et al. [18]. In their large EWAS on current smoking using data from 15,014 samples and the Illumina EPIC array they reported 65,857 differentially methylated CpGs using a false discovery rate correction (FDR < 0.05). From the 24,930 DMPs identified in out meta-analysis 13,023 were also reported by Hoang et al. In Supplementary Table 6 the results from our meta-analyses and the published data are compared. Conversely, 11,907 CpGs that passed the FDR < 0.05 threshold in our meta-analyses were not reported by Hoang et al. (Supplementary Table 7).

For the 11,907 DMPs from our meta-analysis that were not significant in the study by Hoang et al. we performed KEGG pathway analyses and GO functional enrichment. The results are shown in Supplementary Tables 8 and 9. In the KEGG pathway analysis 17 pathways were nominally significant but none passed the threshold of an FDR-corrected p-value < 0.05. In the GO functional enrichment analysis 9 terms were significantly enriched: regulation of small GTPase mediated signal transduction, ruffle, small GTPase-mediated signal transduction, cell surface receptor signaling pathway, cell periphery, anchoring junction, cell junction, plasma membrane region and cell leading edge. We also performed these analyses using all DMPs from the meta-analysis that passed the FDR-corrected p value < 0.05 (Supplementary Tables 10 and 11). In the KEGG pathway analysis 8 significantly enriched pathways were identified: IgSF CAM signaling, Axon guidance, Phospholipase D signaling pathway, Rap1 signaling pathway, Chemokine signaling pathway, cGMP-PKG signaling pathway, Parathyroid hormone synthesis, secretion and action and Integrin signaling. Regarding the GO functional enrichment analysis, we identified 254 significantly enriched terms, the highest-ranking being cell periphery, multicellular organism development, anatomical structure development and system development.

We further investigated the differences in DNA methylation between current smokers and former smokers (Fig. 1B and C). The group of former smokers was split into two based on the time since smoking cessation. Comparing the current smokers with former smokers who had quit smoking more than 10 years before study entry, we found 231 CpGs with significantly different methylation levels after conservative Bonferroni-Holm correction and 2365 CpGs with significantly different methylation levels after FDR correction in the discovery sample. Of these 2365 CpGs 554 were replicated in the replication sample at an FDR-corrected p value < 0.05. Full lists of all significantly different CpGs for both the discovery and the replication sample are given in Supplementary Tables 12 and 13, respectively.

Comparing the DNA methylation of current smokers and short-term quitters (less than 10 years since smoking cessation) resulted in 39 differently methylated CpGs in the discovery sample of which 18 were replicated in the replication sample at an FDR-corrected p value < 0.05. Full lists of all significantly different CpGs for both the discovery and the replication sample are given in Supplementary Tables 14 and 15, respectively. A Venn diagram visualizing the overlap of significant CpGs between the different EWAS analyses in the discovery sample is shown in Supplementary Fig. 18.

**Fig. 2:**
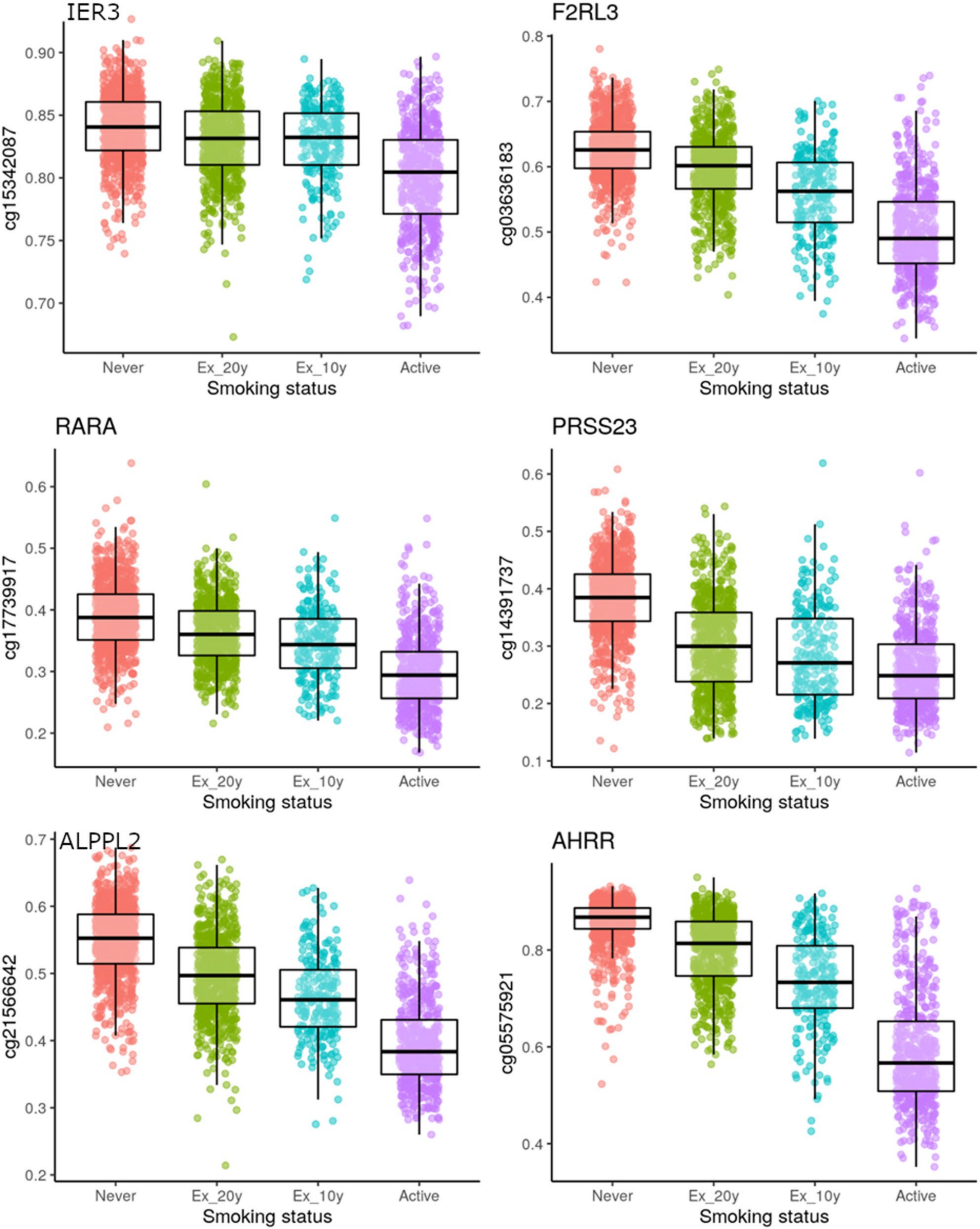
Distribution of methylation beta values of CpGs at the six most significant loci in the different groups analyzed. Beta values of the individual CpGs on the y-axis and smoking status on the x axis. Individual data points are shown as colored dots overlaid by box plots. The line shows the median value and 50% of all values (the 2nd and 3rd quartile) are included in the box

To illustrate the time-dependent changes in DNA methylation we present the distribution of methylation beta values for the leading CpGs from the highest-ranking loci from the comparison of current vs. never smokers stratified for smoking status in Fig. 2. A similar graph showing the distribution of methylation beta values for the leading CpGs color-coded for discovery and replication sample is presented in Supplementary Fig. 19. In analyses stratified by tertiles of hsCRP the association of the DNA methylation changes at the top loci with smoking status was similar in all three tertiles demonstrating robustness with regard to inflammation (Supplementary Table 16). Correlation of the top loci with pack-years are shown in Supplementary Fig. 20.

We also performed EWAS of current smokers vs. never smokers using pack-years as additional variable in the adjustment. Results are presented in Supplementary Table 17. Only 871 differentially methylated CpGs remained significant.

We further performed EWAS of current smokers vs. never smokers using only 10 PCs to adjust for technical artefacts (Supplementary Table 18). This analysis resulted in 12,661 significant DMPs after FDR-correction in the discovery sample.

### Association with mortality and mediation analysis

**Fig. 3:**
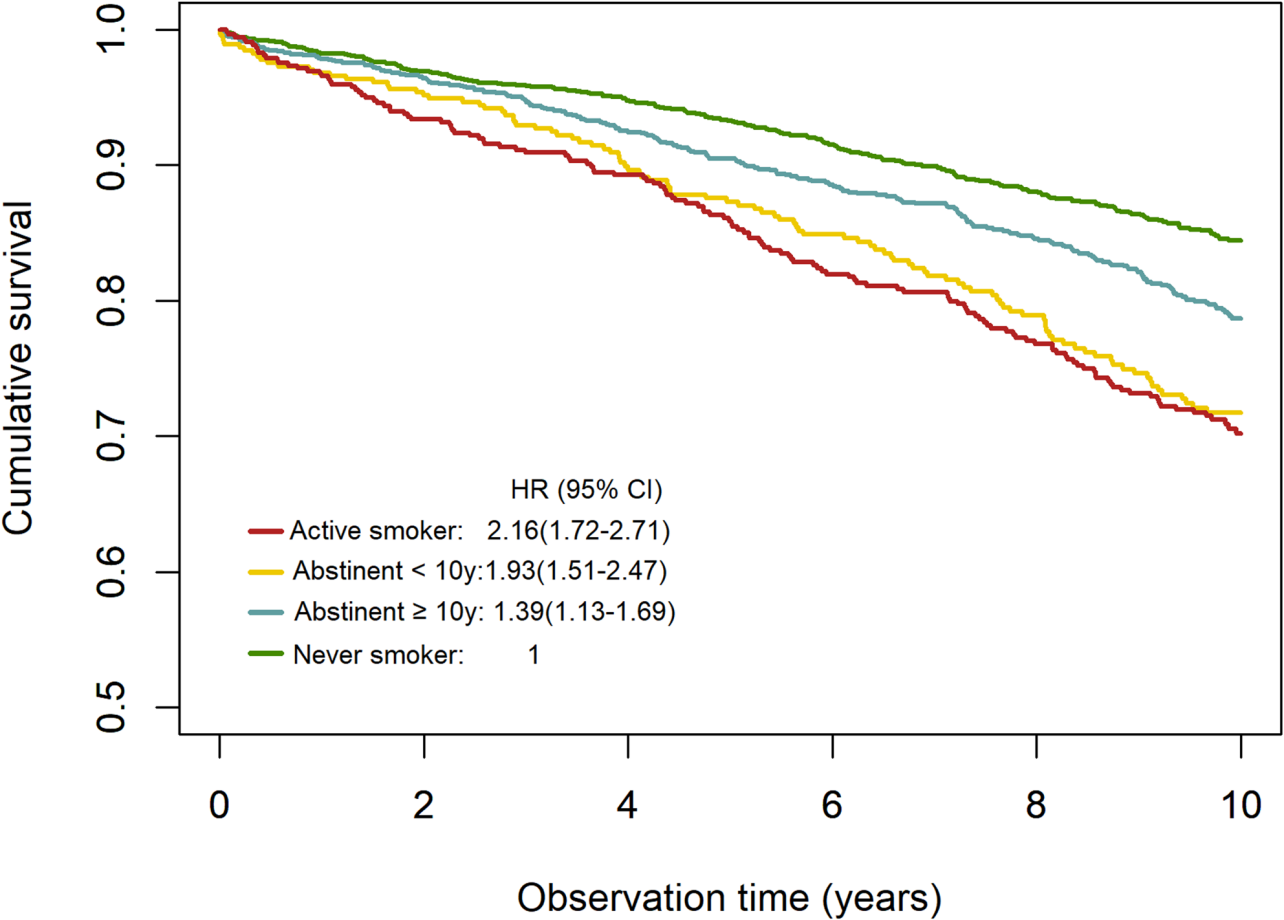
Association of smoking status with all-cause mortality, adjusted for age, sex, BMI and diabetes mellitus.HR: hazard ration; CI: confidence interval

In Cox regression analysis adjusted for age, sex, BMI, hypertension and DM, smoking and former smoking were significantly associated with the risk of all-cause mortality (Fig. 3). We further analysed the association of smoking status with all-cause, cardiovascular and cancer mortality with adjustment for age, sex and, BMI and additional adjustment for DMPs at the six loci that showed the most significant association with active smoking compared to never smokers (Table 5). All DMPs except for cg17739917 at the RARA locus showed significant association with mortality and attenuated the association of active smoking with mortality or even rendered it insignificant. Combining the 5 DMPs that were significantly associated with mortality into a CpG score and using this score as a variable in the Cox regression analyses rendered smoking status completely insignificant. The distribution of the CpG score across smoking categories is shown in Supplementary Fig. 21.

**Table 5:** Association of smoking status and CpGs with mortality

	All-cause mortality* (*N* = 734)		CV mortality* (*N* = 466)		Cancer mortality* (*N* = 106)	
	HR(95%CI)	*P*	HR(95%CI)	*P*	HR(95%CI)	*P*
Never smoker	1		1		1	
Ex-smoker (≥ 10 years)	1.44(1.18–1.75)	< 0.001	1.34(1.05–1.72)	0.018	1.66(0.917–3.01)	0.094
Ex-smoker (< 10 y)	2.02(1.58–2.58)	< 0.001	1.6(1.16–2.20)	0.004	4.26(2.30–7.92)	< 0.001
Active smoker	2.25(1.79–2.82)	< 0.001	1.85(1.39–2.48)	< 0.001	4.71(2.64–8.41)	< 0.001
Never smoker	1		1		1	
Ex-smoker (≥ 10 years)	1.27(1.04–1.57)	0.022	1.25(0.967–1.60)	0.088	1.32(0.718–2.44)	0.369
Ex-smoker (< 10 years)	1.47(1.11–1.95)	0.006	1.31(0.913–1.87)	0.144	2.32(1.13–4.75)	0.022
Active smoker	1.28(0.922–1.76)	0.142	1.29(0.853–1.94)	0.230	1.61(0.70–3.72)	0.261
cg05575921 (AHRR)	0.739(0.656–0.831)	< 0.001	0.821(0.705–0.955)	0.010	0.568(0.423–0.763)	< 0.001
Never smoker	1		1		1	
Ex-smoker (≥ 10 years)	1.25(1.02–1.54)	0.032	1.22(0.943–1.57)	0.132	1.42(0.769–2.61)	0.263
Ex-smoker (< 10 years)	1.53(1.17–2.17)	0.002	1.30(0.917–1.83)	0.142	3.08(1.55–6.10)	0.001
Active smoker	1.40(1.05–1.88)	0.022	1.30(0.90–1.88)	0.162	2.72(1.28–5.77)	0.009
cg21566642 (ALPPL2)	0.765(0.692–0.847)	< 0.001	0.817(0.719–0.928)	0.002	0.732(0.559–0.957)	0.023
Never smoker	1		1		1	
Ex-smoker (≥ 10 years)	1.34(1.09–1.64)	0.005	1.28(0.999–1.64)	0.051	1.45(0.793–2.64)	0.228
Ex-smoker (< 10 years)	1.66(1.28–2.17)	< 0.001	1.4(0.992–1.97)	0.055	2.92(1.49–5.73)	0.002
Active smoker	1.63(1.23–2.16)	0.001	1.48(1.04–2.11)	0.029	2.53(1.24–5.20)	0.011
cg03636183 (F2RL3)	0.819(0.743–0.903)	< 0.001	0.87(0.769–0.984)	0.027	0.681(0.53–0.875)	0.003
Never smoker	1		1		1	
Ex-smoker (≥ 10 years)	1.45(1.19–1.78)	< 0.001	1.38(1.08–1.77)	0.010	1.59(0.873–2.89)	0.130
Ex-smoker (< 10 years)	2.08(1.61–2.67)	< 0.001	1.7(1.23–2.36)	0.001	3.86(2.04–7.30)	< 0.001
Active smoker	2.39(1.86–3.07)	< 0.001	2.10(1.53–2.89)	< 0.001	3.87(2.01–7.43)	< 0.001
cg17739917 (RARA)	1.05(0.96–1.15)	0.282	1.11(0.992–1.24)	0.069	0.849(0.666–1.08)	0.189
Never smoker	1		1		1	
Ex-smoker (≥ 10 years)	1.21(0.984–1.50)	0.070	1.23(0.952–1.59)	0.114	1.11(0.591–2.07)	0.753
Ex-smoker (< 10 years)	1.57(1.20–2.04)	0.001	1.39(0.992–1.96)	0.055	2.33(1.17–4.62)	0.015
Active smoker	1.62(1.25–2.10)	< 0.001	1.55(1.12–2.15)	0.008	2.21(1.13–4.34)	0.020
cg14391737 (PRSS23)	0.771(0.703–0.847)	< 0.001	0.869(0.775–0.974)	0.016	0.543(0.416–0.708)	< 0.001
Never smoker	1		1		1	
Ex-smoker (≥ 10 years)	1.39(1.14–1.70)	0.001	1.31(1.02–1.67)	0.033	1.63(0.898–2.96)	0.109
Ex-smoker (< 10 years)	1.89(1.47–2.42)	< 0.001	1.50(1.09–2.07)	0.013	4.11(2.20–7.67)	< 0.001
Active smoker	1.86(1.45–2.38)	< 0.001	1.56(1.14–2.14)	0.006	4.27(2.27–8.02)	< 0.001
cg15342087 (IER3)	0.852(0.787–0.922)	0.000	0.863(0.781–0.954)	0.004	0.92(0.75–1.13)	0.421
Never smoker	1		1		1	
Ex-smoker (≥ 10 years)	1.20(0.972–1.48)	0.089	1.20(0.925–1.55)	0.173	1.20(0.645–2.23)	0.565
Ex-smoker (< 10 years)	1.35(1.02–1.79)	0.038	1.22(0.852–1.76)	0.274	2.07(0.996–4.28)	0.051
Active smoker	1.14(0.824–1.57)	0.431	1.18(0.785–1.77)	0.426	1.42(0.614–3.29)	0.412
**CpG score**	1.43(1.27–1.61)	< 0.001	1.27(1.10–1.47)	0.001	1.88(1.39–2.53)	< 0.001

* Cox regession analyses of smoking status and individual CpGs or CpG score adjusted for age, sex and BMI. HR: hazard ratio; CI: confidence interval

We also performed a mediation analysis to examine whether the changes in DNA methylation that are associated with smoking might mediate the effect of smoking on all-cause mortality (Table 6).

**Table 6:** Mediation analysis showing the natural direct effect of smoking status, the natural indirect effect through the mediator (CpG score, per 1-SD increase) and the total effect on all-cause mortality

Effect type	Hazard ratio	95% confidence interval	*p*-value
Total Effect (TE)	1.45	1.29–1.62	2.32E-10
Pure Natural Direct Effect (PNDE)	1.10	0.98–1.24	1.07E-01
Pure Natural Indirect Effect (PNIE)	1.38	1.23–1.55	6.60E-08
Controlled Direct Effect (CDE)*	1.07	0.96–1.19	2.22E-01

In the mediator model, smoking status (modeled as an ordinal variable with four categories: never smokers, ex-smokers > = 10 years, ex-smokers < 10 years and current smokers) was strongly associated with DNA methylation levels. Per one-category increase in smoking status, methylation increased by 0.67 units (β = 0.669, SE = 0.012, *p* < 2 × 10⁻¹⁶), independent of age and sex. The model explained 62% of the variance in methylation levels (adjusted R² = 0.623).

In Cox proportional hazards models including an exposure–methylation interaction term, DNA methylation was significantly associated with higher risk of all-cause mortality with a HR (95%CI) of 1.62 (1.36–1.93; *p* < 0.001) per unit increase). The direct association between smoking status and the outcome was not statistically significant after inclusion of methylation in the model (HR 1.07 (0.96–1.19)). The exposure–methylation interaction term was of borderline statistical significance (HR 0.92 (0.85–1.00); *p* = 0.056), suggesting possible effect modification. In counterfactual mediation analyses, each one-category increase in smoking status was associated with a 45% higher hazard of all-cause mortality (total effect 1.45 (1.29–1.62). The natural indirect effect through DNA methylation corresponded to a HR of 1.38 (1.23–1.55), whereas the natural direct effect was not statistically significant (HR 1.05 (0.94–1.16)) (Table 6). The proportion mediated on the log-hazard scale was estimated at 77%.

These findings are consistent with substantial mediation of the smoking–outcome association through DNA methylation, although direct effects independent of methylation were not statistically evident in this model. Results remained stable after further adjustment for diabetes mellitus, hypertension, BMI and Houseman cell types (Supplementary Table 19).

The CpG score showed a correlation with leucocytes and hsCRP as markers of increased inflammation while it was inversely correlated with high density lipoprotein-cholesterol HDL-C levels (Supplementary Fig. 22). We also compared the predictive performance of our CpG score with CpG scores based on 2 and 10 CpGs that had been published previously [24, 25]. Both previously published CpG scores included cg06126421 near the IER gene that was included in the 450 array but not in out EPIC array. We therefore substituted this CpG with the highest ranking in our EWAS at that region (cg15342087). Furthermore, cg23665802 near MIR19A was also not available in our dataset and was omitted from the 10-CpG score. The 10-CpG score was significantly better in predicting all-cause mortality than our CpG score (Supplementary Table 20).

## Discussion

In our study, we found 1071 CpGs that had significantly different methylation levels after Bonferroni-Holm correction and 14,403 CpGs that had significantly different methylation levels after FDR correction when comparing active smokers with never smokers. Our most significant loci near the genes AHRR, ALPPL2 (described in previous publications as region 2q37.1), F2RL3, RARA (retinoic acid receptor α), PRSS23 (serine protease 23), and IER3 have been associated with smoking in previous EWAS [7, 26–28] and were confirmed in internal validation using the replication sample. These genes are associated with cell proliferation and thus with the development of cancer and cardiovascular death. For most of the gene loci, a decrease in the degree of methylation was observed for the active smokers as compared to ex-smokers and never smokers. Even in ex-smokers who had stopped smoking more than 10 years ago, the degree of methylation for some of the loci was still lower than in study participants who had never smoked.

The AHRR gene encodes a repressor for the aryl hydrocarbon receptor (AHR), which plays a role in dioxin toxicity and in the regulation of cell growth and differentiation. AHR is activated by exogenous as well as by endogenous ligands and is translocated to the nucleus where is forms a complex with AHR nuclear translocator (ARNT). By binding to dioxin-response-elements this complex activates the transcription of a number of genes, one of the most important being CYP1A1 [29]. As a sensor for environmental stimuli it plays an important role in homoeostasis, i.a. in the gut, and constitutes an important regulator of the immune system [29]. Hypomethylation of cg05575921 at the AHRR gene has been consistently observed in multiple tissues, including blood, saliva, and lung [30] and has also been associated with smoking-induced morbidity and mortality [31].

The ALPPL2 gene (a.k.a. ALPG, alkaline phosphatase, germ cell) encodes a membrane-bound alkaline phosphatase localized in testis, thymus, and certain germ cell tumors. It has been associated with cancer [32] The F2R-like thrombin or trypsin receptor 3 (F2RL3) is expressed in a variety of tissues with the highest expression in lungs, pancreas, thyroid, testes, small intestine and platelets and may play a role in platelet activation. Serine protease 23, encoded by the PRSS23 gene, is particularly highly expressed in the gallbladder and urinary bladder. The RARA gene encodes retinoic acid receptor α, a transcription factor associated with regulation of development, differentiation, apoptosis, granulopoiesis, and transcription of clock genes. IER3 protects cells from Fas - or TNFα-induced apoptosis. The general description of genes was adopted from GeneCards [33].

For all gene loci, a decrease in the level of methylation was observed starting from never smokers to ex-smokers to active smokers. Even in the ex-smokers who had already quit smoking more than 10 years ago, the methylation level was still significantly (PRSS23, AHRR, ALPPL2) or at least in trend (F2RL3, RARA, IER3) lower compared to the study participants who never smoked.

There is a risk of bias regarding the self-reported smoking and the reported time point of smoking cessation that we would like to mention. Although we used cotinine measurements to reclassify self-reported non-smoking participants into the group of current smokers when their cotinine value exceeded 15 µg/l we cannot exclude the possibility that former smokers falsely reported to be never smokers or that they supplied an incorrect time point regarding smoking cessation. However, we are confident that this will not severly influence our results given our fairly high sample size.

The existence of long-lasting changes in the methylation status of leukocytes has been noted, in some studies. Guida et al. classified differentially methylated CpGs between smokers and never smokers into two groups: in the first group, the methylation level “normalized” after smoking cessation (range of 0–35 years) and returned to levels comparable to those of never smokers, while the methylation level of CpGs in the second group remained altered even more than 35 years after smoking abstinence [27]. The authors postulated that this difference could be explained, at least in essence, by the extent of the change in methylation induced by smoking. For those CpGs in which the greatest changes in methylation were observed as a result of smoking, these changes also persisted longer than for CpGs in which the change had been smaller.

Usually, CpGs with significant differences in DNA methylation were hypomethylated in current smokers. From the 24,930 DMPs from our meta-analysis 17,711 (71%) showed hypomethylation. Amongst the hypermethylated loci was a.o. a DMP at the SELL (selectin L) gene. The SELL gene encodes a cell surface adhesion molecule that belongs to a family of adhesion/homing receptors. Hypermethylation at this locus may at least partly explain the lower levels of L-selectine in active smokers that we observed in a previous study [34].

In addition, in our meta-analysis of discovery and replication samples we identified 11,907 novel CpG sites (Supplementary Table 7) that have not been listed in a recently published large EWAS on smoking [18]. Pathway enrichment analysis revealed significant overrepresentation of Gene Ontology terms related to small GTPase-mediated signal transduction, membrane-associated signaling, and cytoskeletal organization for the novel loci. Enriched categories included regulation of small GTPase signaling, membrane ruffling, cell leading edge, anchoring junctions, and cell surface receptor signaling pathways. Collectively, these pathways converge on the regulation of actin cytoskeleton dynamics and cell–cell interactions at the plasma membrane. These findings suggest that these novel smoking-associated epigenetic alterations may preferentially affect signaling pathways controlling cytoskeletal remodeling and cell adhesion, processes central to inflammatory cell trafficking and endothelial dysfunction.

We could further demonstrate that the changes in the DNA methylation level induced by smoking can be regarded as significant mediators of the strong association of smoking status and mortality, especially cancer-related and cardiovascular mortality.

DNA methylation at the F2RL3 locus has been associated with mortality in the past. Zheng et al. demonstrated a strong association of a lower methylation intensity at F2RL3 with mortality after adjustment for multiple covariates including smoking [35] and a more recent study estimated DNA methylation at the F2RL3 locus mediated 34% of the smoking effect on increased risk of myocardial infarction [36] Zhang et al. showed a dose–response relationship of current and lifetime smoking exposure and time since smoking cessation with consistent site-specific methylation at CpGs located in AHRR, F2RL3, 2q37.1 (cg21566642, cg01940273), and 6p21.22 (cg06126421) [24]. They further showed that methylation at seven CpGs was also associated with mortality outcomes and that a score based on methylation at the top two CpGs (cg05575921 at AHRR and cg06126421 at 6p21.33) provided very strong associations with all-cause, cardiovascular, and cancer mortality.

In our study, we combined 5 DMPs that were significantly associated with mortality into a CpG score and using this score as a variable in the Cox regression analyses rendered smoking status insignificant.

In the adjusted mediation model, the natural direct effect was no longer statistically significant, whereas the indirect effect through DNA methylation remained robust. Our findings are therefore in line with previously published research. This pattern is consistent with a predominantly methylation-mediated pathway linking smoking to all-cause mortality. However, the absence of a statistically significant direct effect should not be interpreted as definitive evidence of complete mediation. The direct effect estimate was small and imprecisely estimated, and mediation results rely on assumptions including the absence of unmeasured confounding of the mediator–outcome relationship. Moreover, the inclusion of an exposure–mediator interaction implies that direct and indirect effects are conditional on model specification and covariate values.

We compared our CpG score with the 2-CpG score described above as well as with an extended score based on 10 CpGs [25]. Our CpG score included the two loci from Zhang et al. and did not perform significantly better in the prediction of all-cause mortality than the smaller score based on 2 CpGs. The 10 CpG score outperformed our smaller score with an AUC (95% CI) of 0.632 (0.607–0.656) compared to 0.578 (0.553–0.602). However, this score was specifically designed for the prediction of mortality while our intention was to analyse whether DNA methylation changes mediate the effect of smoking on mortality.

Smoking only a few cigarettes per day measurably alters methylation, particularly at the AHRR gene. Such epigenetic changes can also be detected with light cigarettes and occasional smoking [37]. Due to the consistently shown association of smoking-induced DNA methylation changes with mortality it seems that there exists no threshold below which smoking would be ‘harmless’, emphasising the importance of smoking prevention.

## Limitations and strengths

The following limitations must be acknowledged. First, the cross-sectional design of our study restricts our ability to infer temporal dynamics or causal relationships between smoking and DNA methylation. Longitudinal data would be needed to distinguish transient from persistent epigenetic alterations. Second, methylation was assessed in whole blood, which—while readily accessible—may not fully capture tissue-specific effects relevant to smoking-associated diseases such as cancer or cardiovascular pathology. Third, smoking-induced shifts in immune cell composition, which themselves alter methylation profiles, may act as confounders. However, we applied the widely used Houseman algorithm to estimate white blood cell subtypes. Fourth, as LURIC only included patients that had been referred for coronary angiography our results are are not generalizable to the general population. Fifth, the time point of smoking cessation was reported by the study participants in a questionnaire at study entry and a misclassification of former smokers due to falsely reported time point of smoking cessation is possible.

A significant strength of our study is using a large, well-characterised patient cohort with extensive clinical data and a genome-wide DNA methylation profile generated via the Illumina EPIC BeadChip platform. This allowed for a comprehensive interrogation of methylation patterns. We replicated key findings across independent subsamples, lending robustness, internal validity to our results. This underscores the utility of our methylation signatures as stable biomarkers of cumulative tobacco exposure, which is particularly relevant to former smokers, in whom traditional exposure metrics often lack precision.

Future studies should apply high-throughput techniques in well-characterised longitudinal cohorts to clarify how smoking-induced DNA methylation changes contribute to cardiovascular mortality and related adverse outcomes.

Parallel assessment of electronic cigarettes and heated tobacco products is needed to determine their epigenetic effects relative to conventional smoking. Comparative profiling across exposure types may identify distinct molecular signatures linked to cardiovascular risk, informing proportionate regulatory responses. Expanding research to younger and underrepresented populations will improve the validity and equity of methylation-based prediction models. Incorporating epigenetic biomarkers into cardiovascular risk assessment and prevention strategies offers a timely opportunity to advance precision public health.

## Conclusion

In summary, our findings substantiate and expand upon existing evidence that tobacco smoking induces widespread, exposure-dependent alterations in DNA methylation, many of which persist for years following cessation. These epigenetic modifications, carry potential implications for immune regulation, inflammation, and carcinogenesis. The results advocate integrating DNA methylation markers into refined risk stratification models and long-term surveillance strategies, particularly in preventive cardiology and oncology, which bridge molecular epidemiology and personalised prevention. Knowing their personal DNA methylation score and the risk associated with it may provide a further incentive for smokers to quit smoking.

## Supplementary Information

Supplementary Material 1.

Supplementary Material 2.

## Data Availability

The data analyzed in this study is subject to the following licenses/restrictions: Due to the articles of Ludwigshafen Risk and Cardiovascular Health (LURIC) Study GmbH, which needs to acknowledge the German Data Protection Act and the consent given by the study participants, data cannot be released to the public domain. Interested researchers are invited to address their request or proposal to access the dataset to Kai Grunwald (kai.grunwald@weitnauer.net) or to the principal investigator of the LURIC study WM (winfried.maerz@luric-online.de).
