# Supplementary material for: Changes of DNA methylation in smokers and ex-smokers referred for coronary angiography. Results from the LURIC study
Source: Clin Epigenetics. 2026 Apr 4;18:68. doi: 10.1186/s13148-026-02118-9 (PMC13088500; doi:10.1186/s13148-026-02118-9)
Supplement: Supplementary file 1 — Supplementary Material 1. [file 13148_2026_2118_MOESM1_ESM.docx]

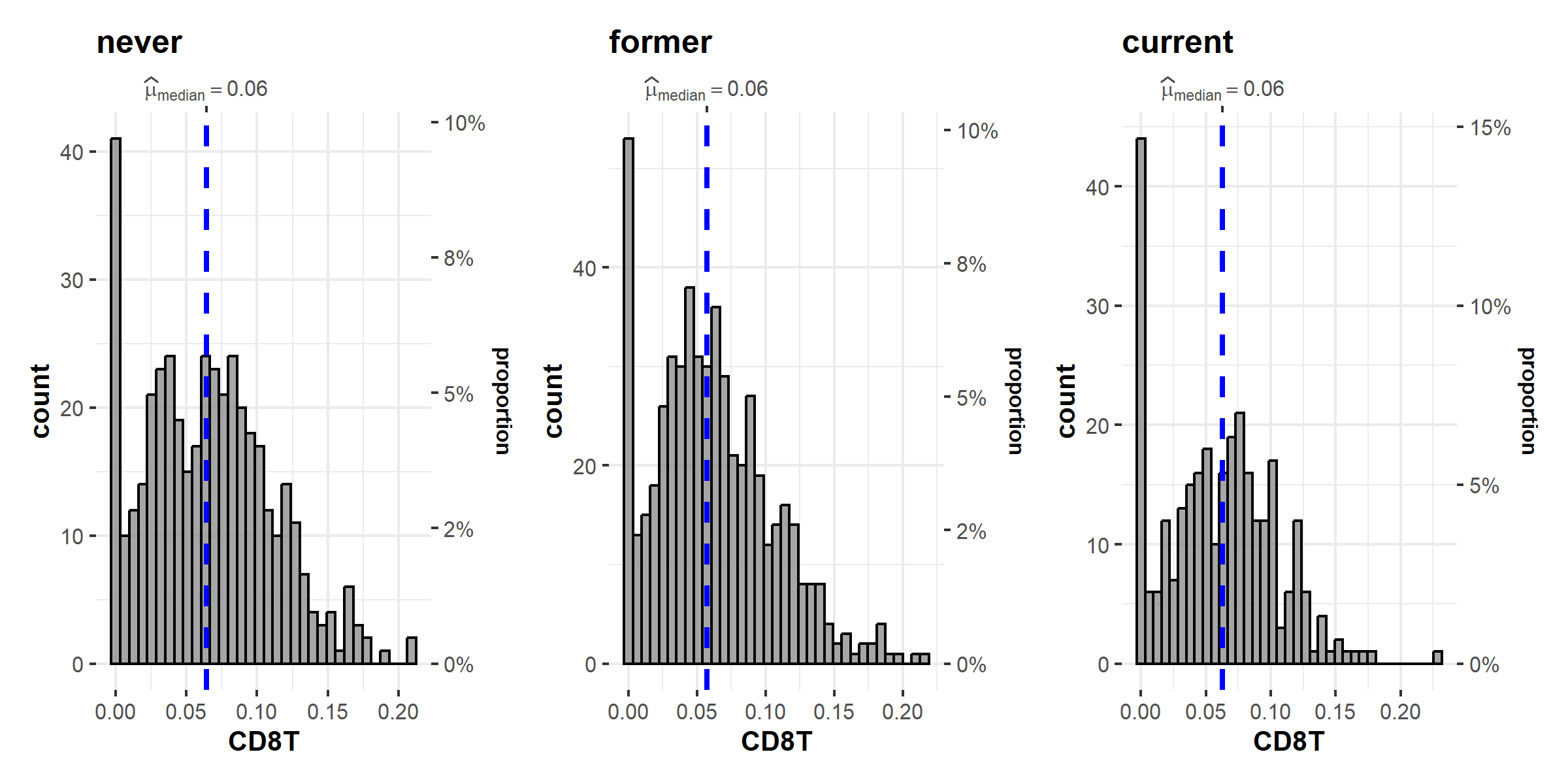


**Supplementary Figure 1:** Distribution of CD8T cells calculated by the Houseman algorithm and stratified by smoking status in the discovery sample


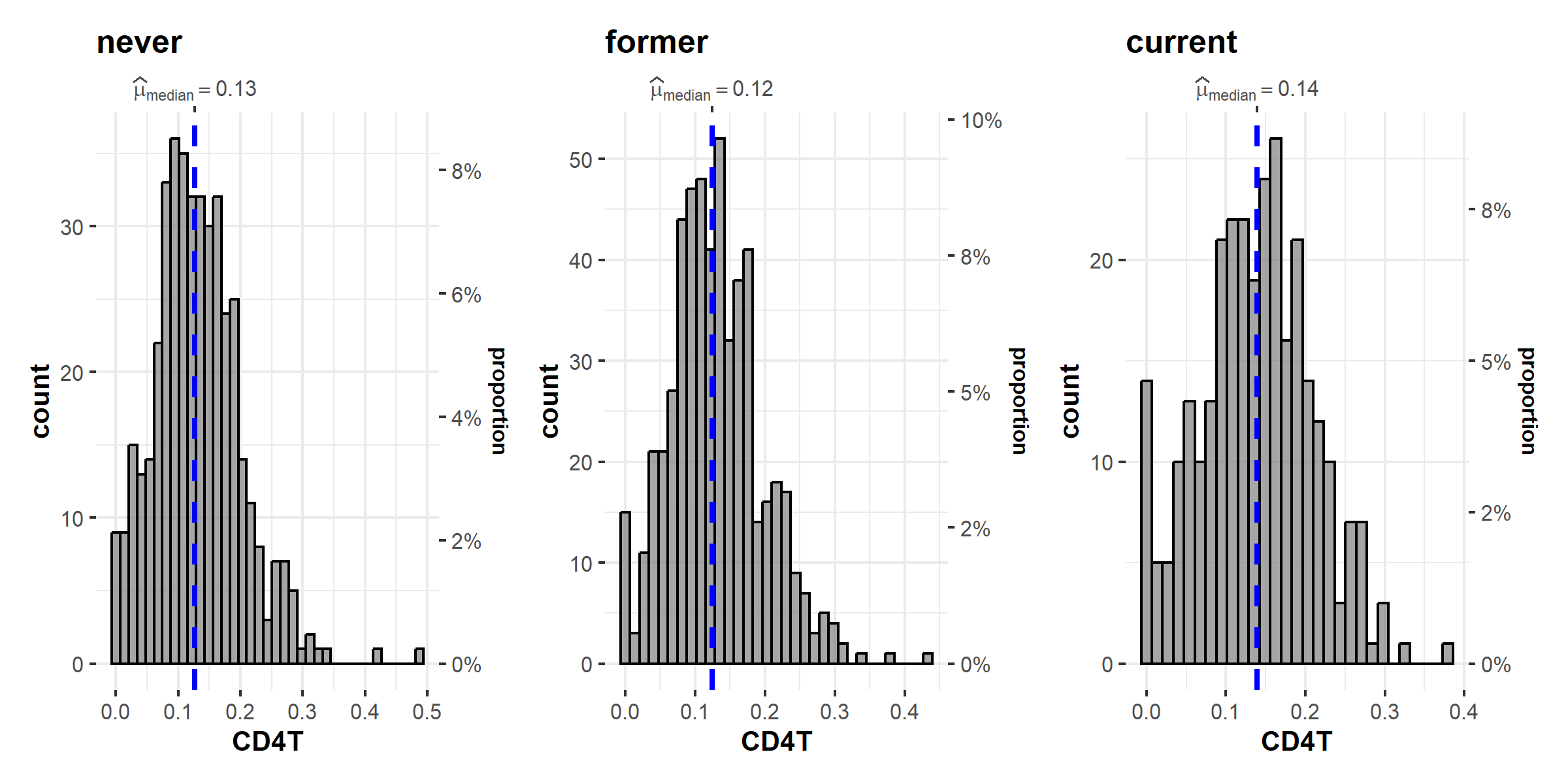


**Supplementary Figure 2:** Distribution of CD4T cells calculated by the Houseman algorithm and stratified by smoking status in the discovery sample


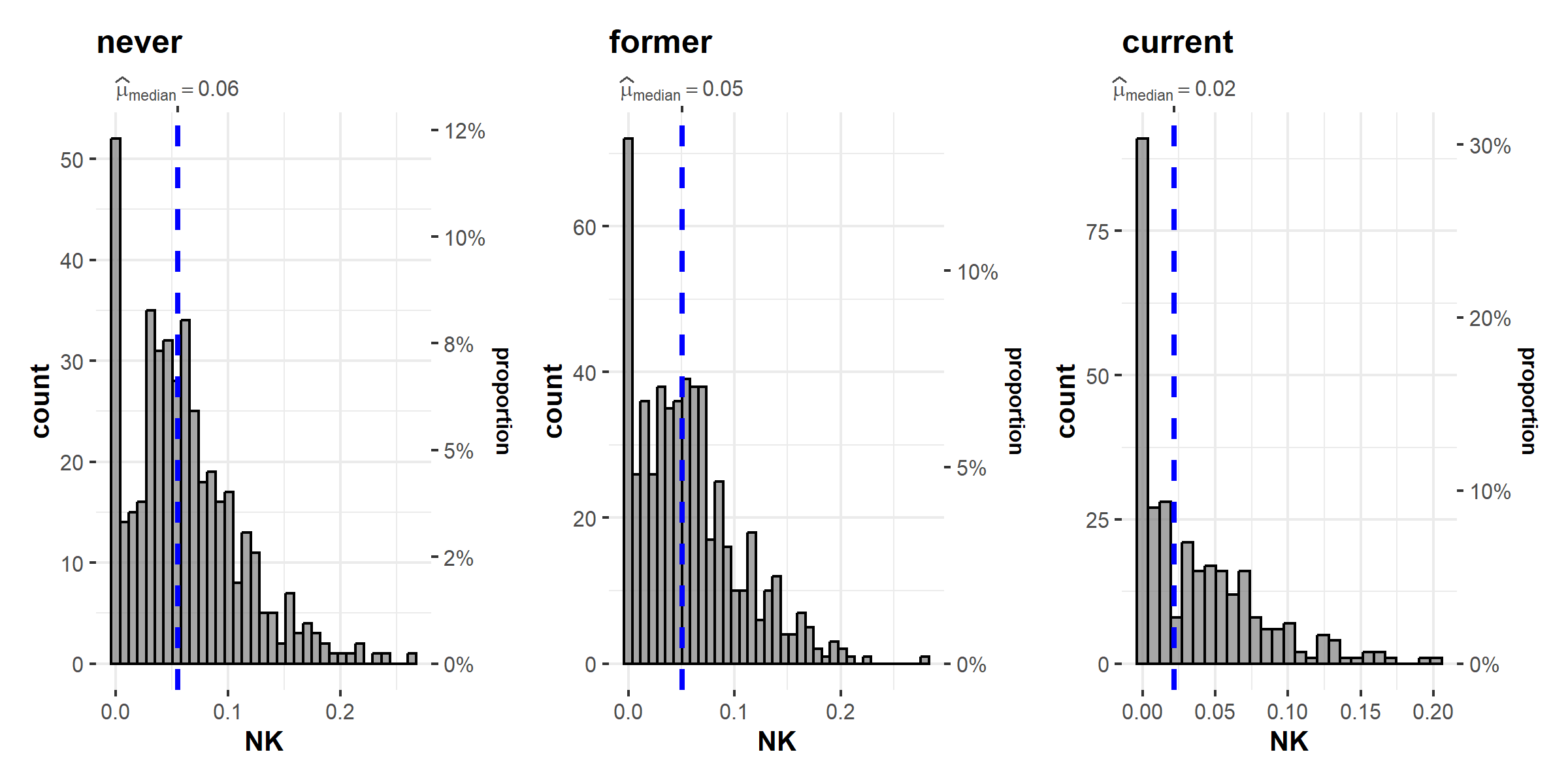


**Supplementary Figure 3:** Distribution of NK cells calculated by the Houseman algorithm and stratified by smoking status in the discovery sample


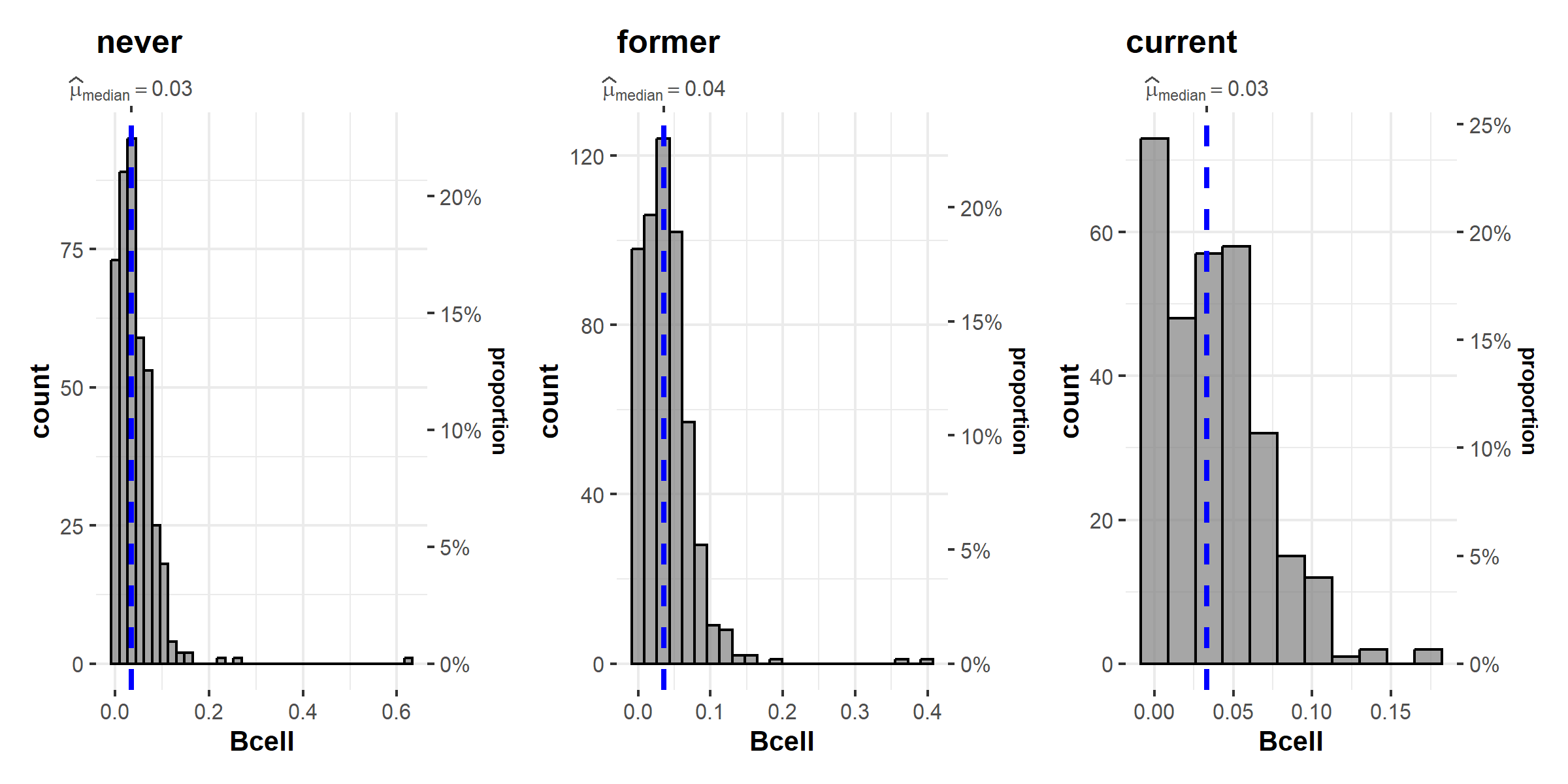


**Supplementary Figure 4:** Distribution of Bcells calculated by the Houseman algorithm and stratified by smoking status in the discovery sample


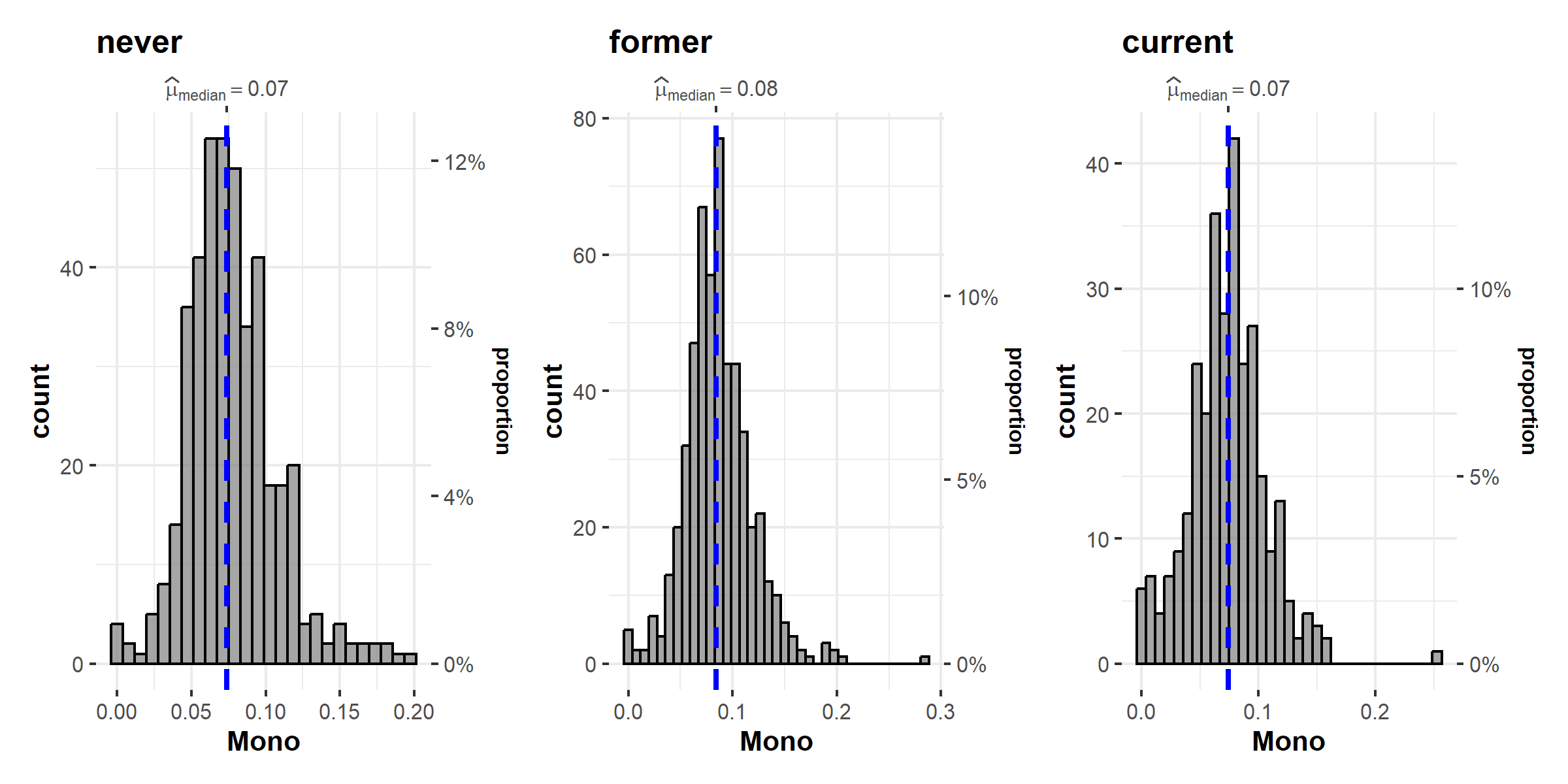


**Supplementary Figure 5:** Distribution of monocytes calculated by the Houseman algorithm and stratified by smoking status in the discovery sample


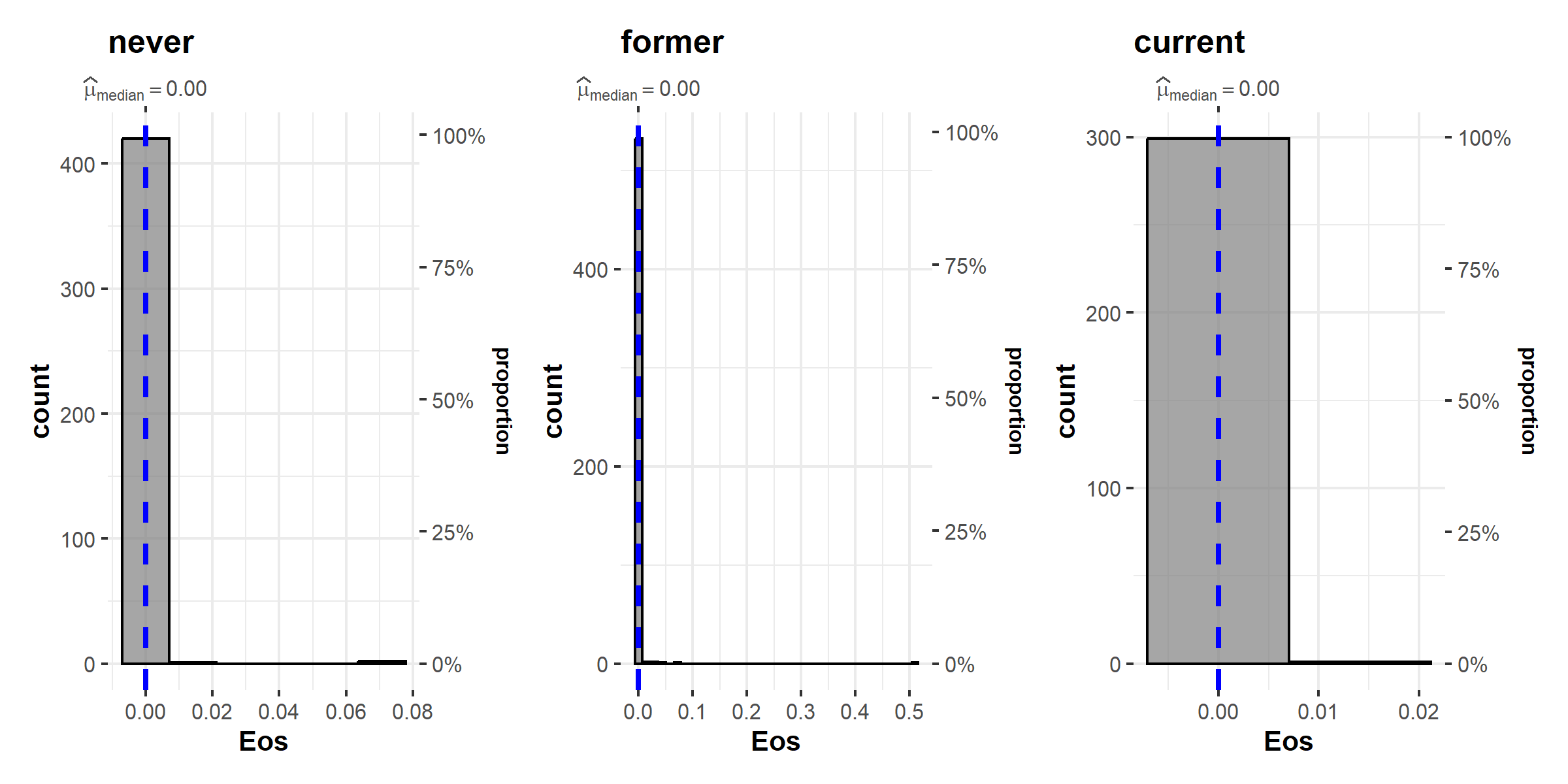


**Supplementary Figure 6:** Distribution of eosinophiles calculated by the Houseman algorithm and stratified by smoking status in the discovery sample


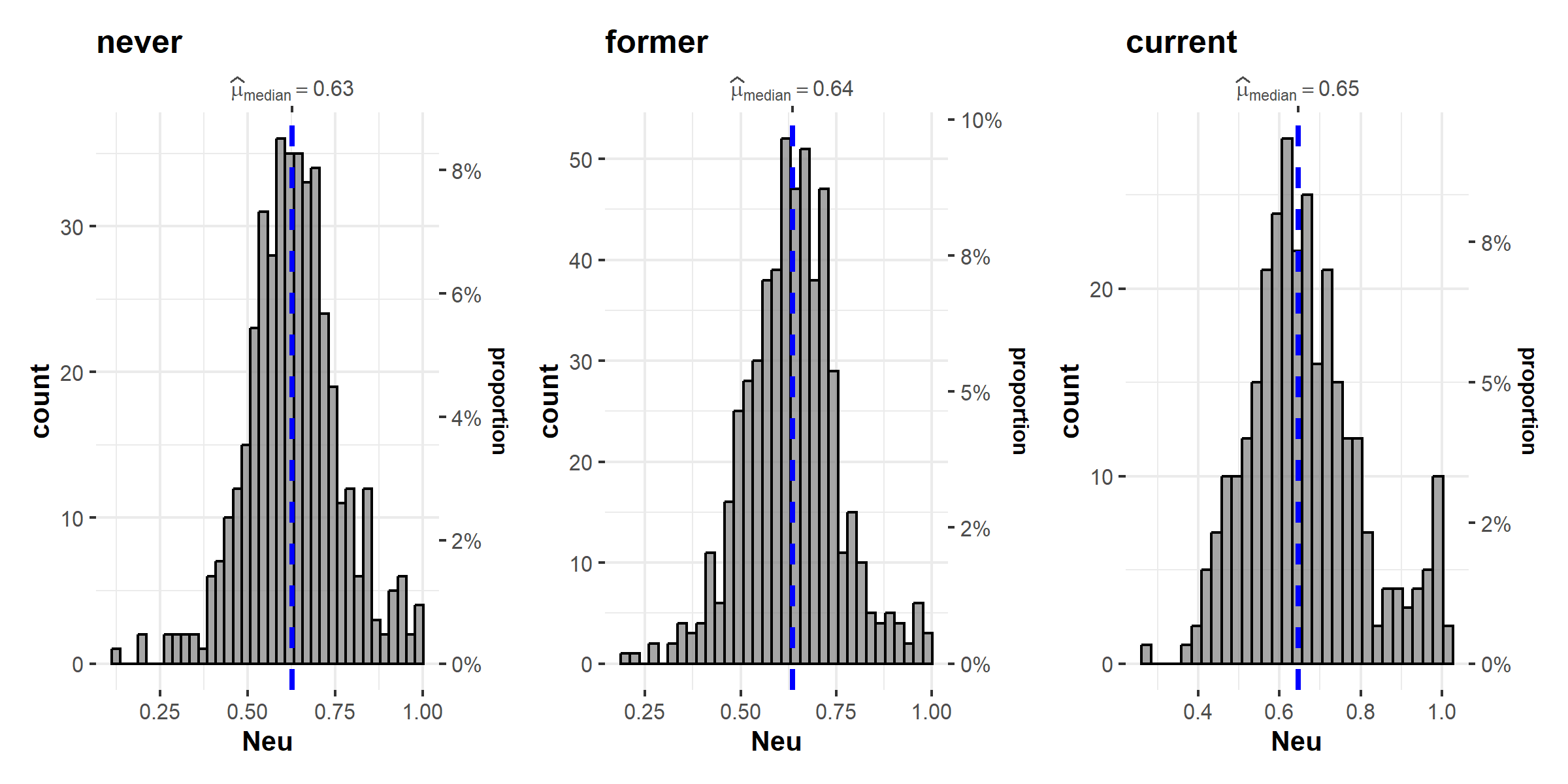


**Supplementary Figure 7:** Distribution of neutrophiles calculated by the Houseman algorithm and stratified by smoking status in the discovery sample


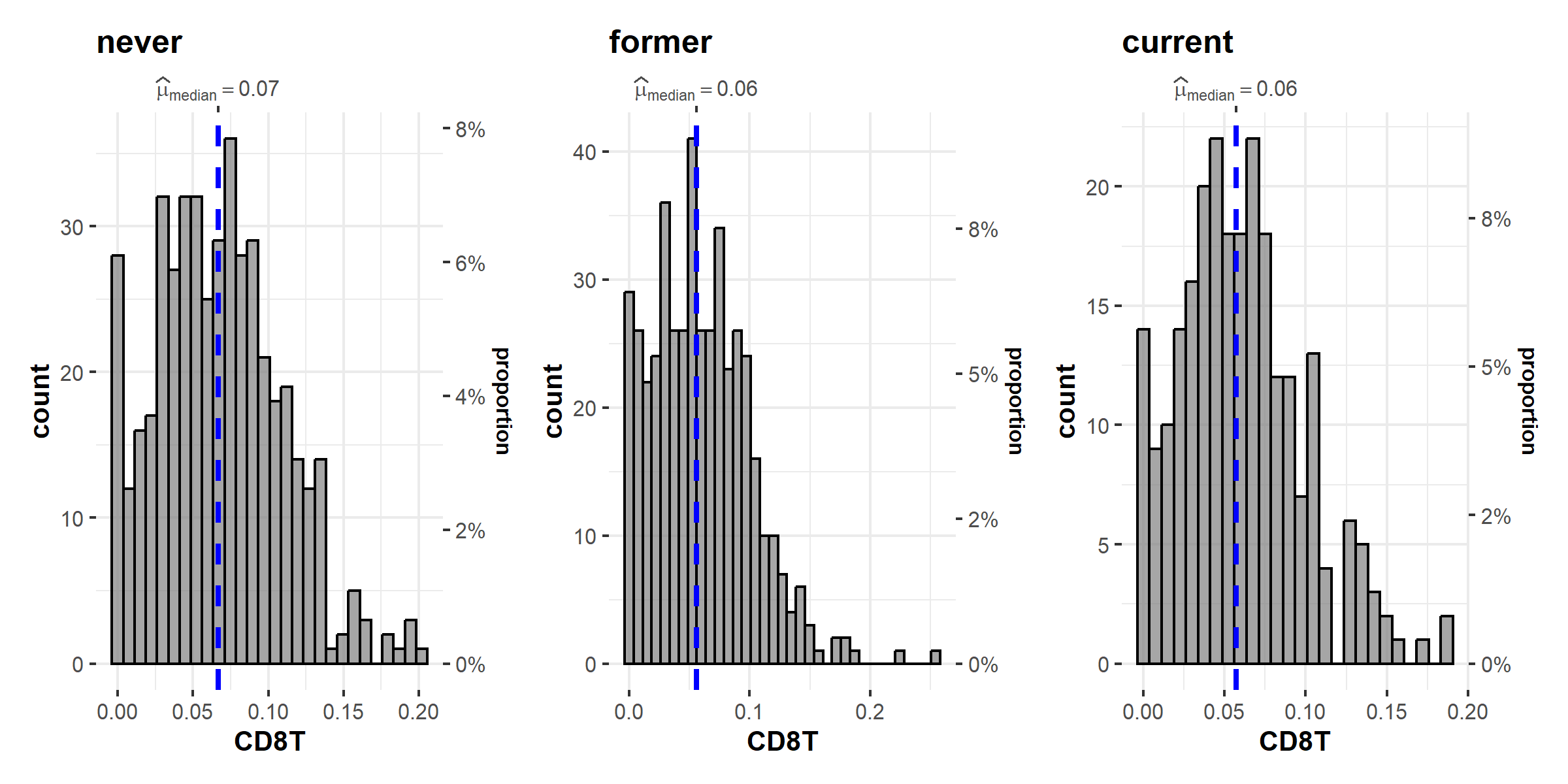


**Supplementary Figure 8:** Distribution of CD8T cells calculated by the Houseman algorithm and stratified by smoking status in the replication sample


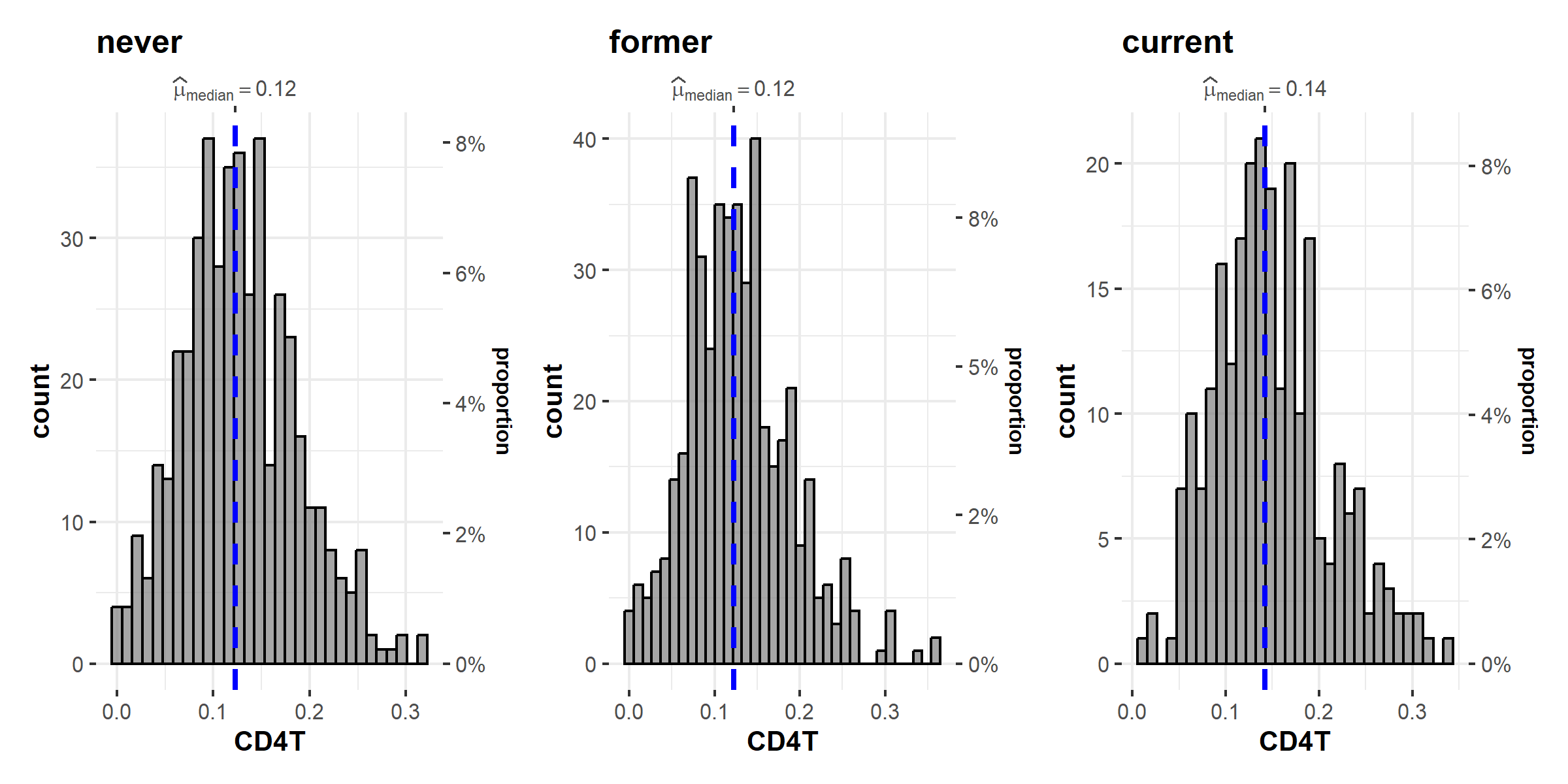


**Supplementary Figure 9:** Distribution of CD4T cells calculated by the Houseman algorithm and stratified by smoking status in the replication sample


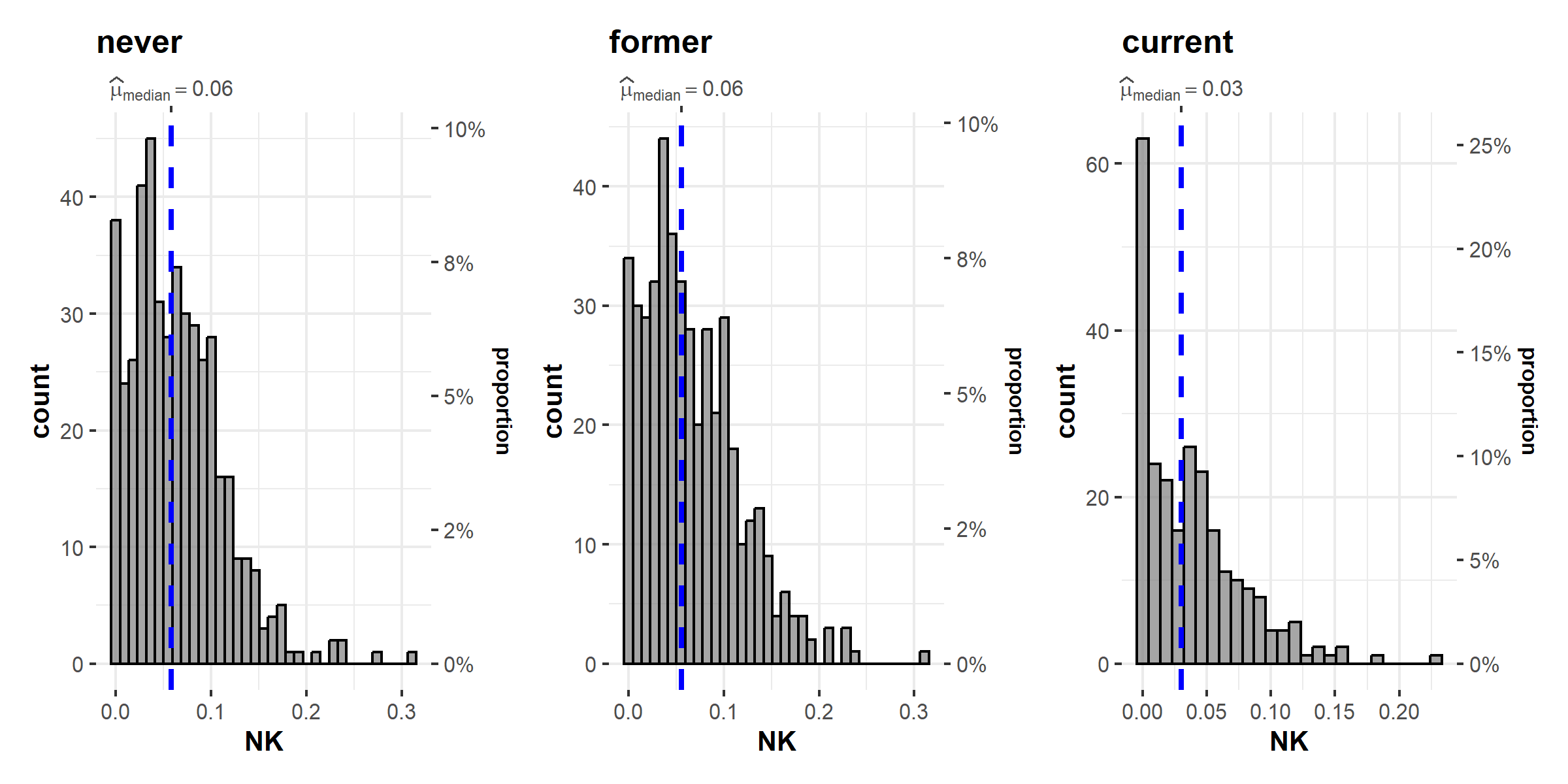


**Supplementary Figure 10:** Distribution of NK cells calculated by the Houseman algorithm and stratified by smoking status in the replication sample


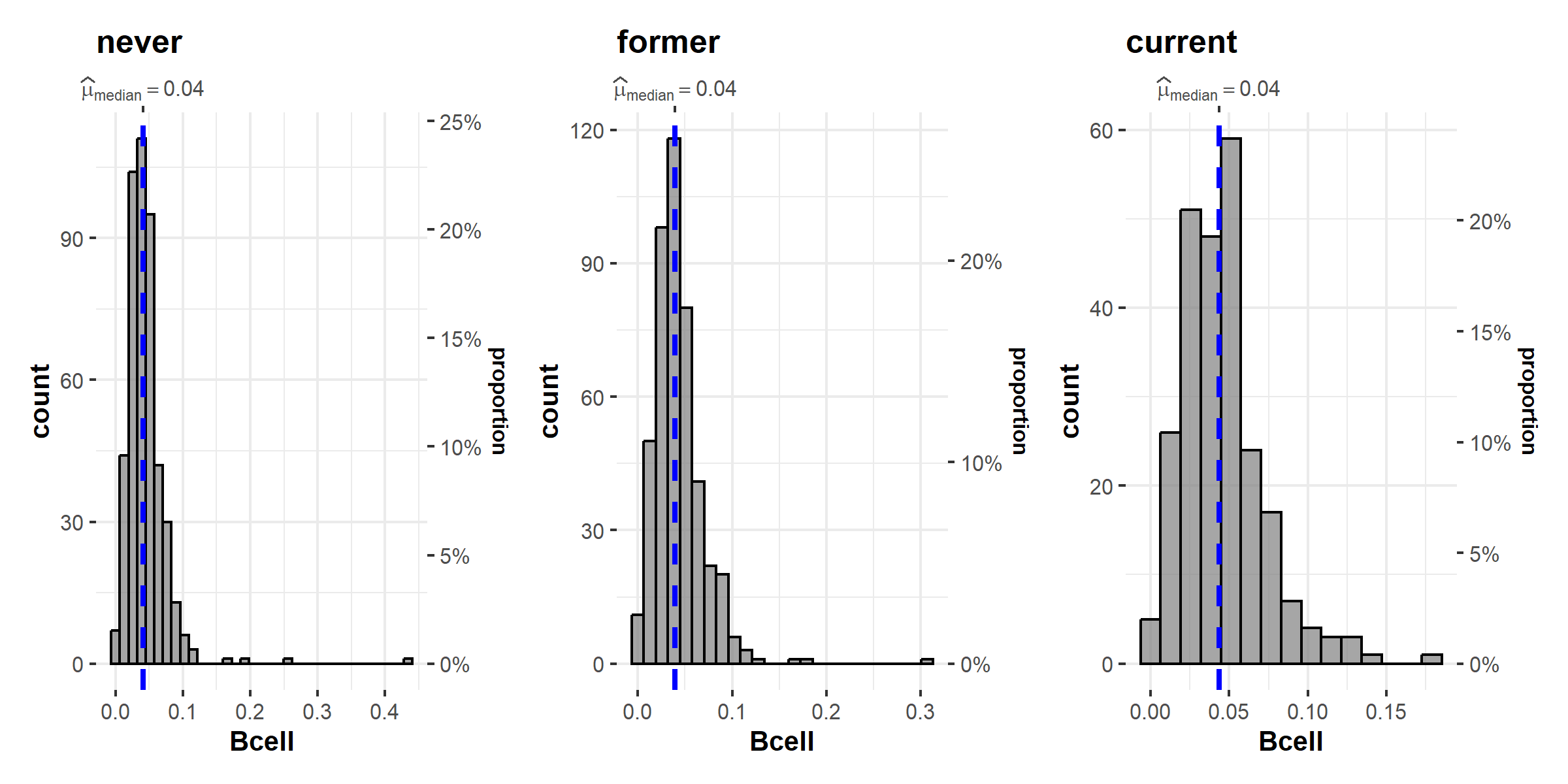


**Supplementary Figure 11:** Distribution of Bcells calculated by the Houseman algorithm and stratified by smoking status in the replication sample


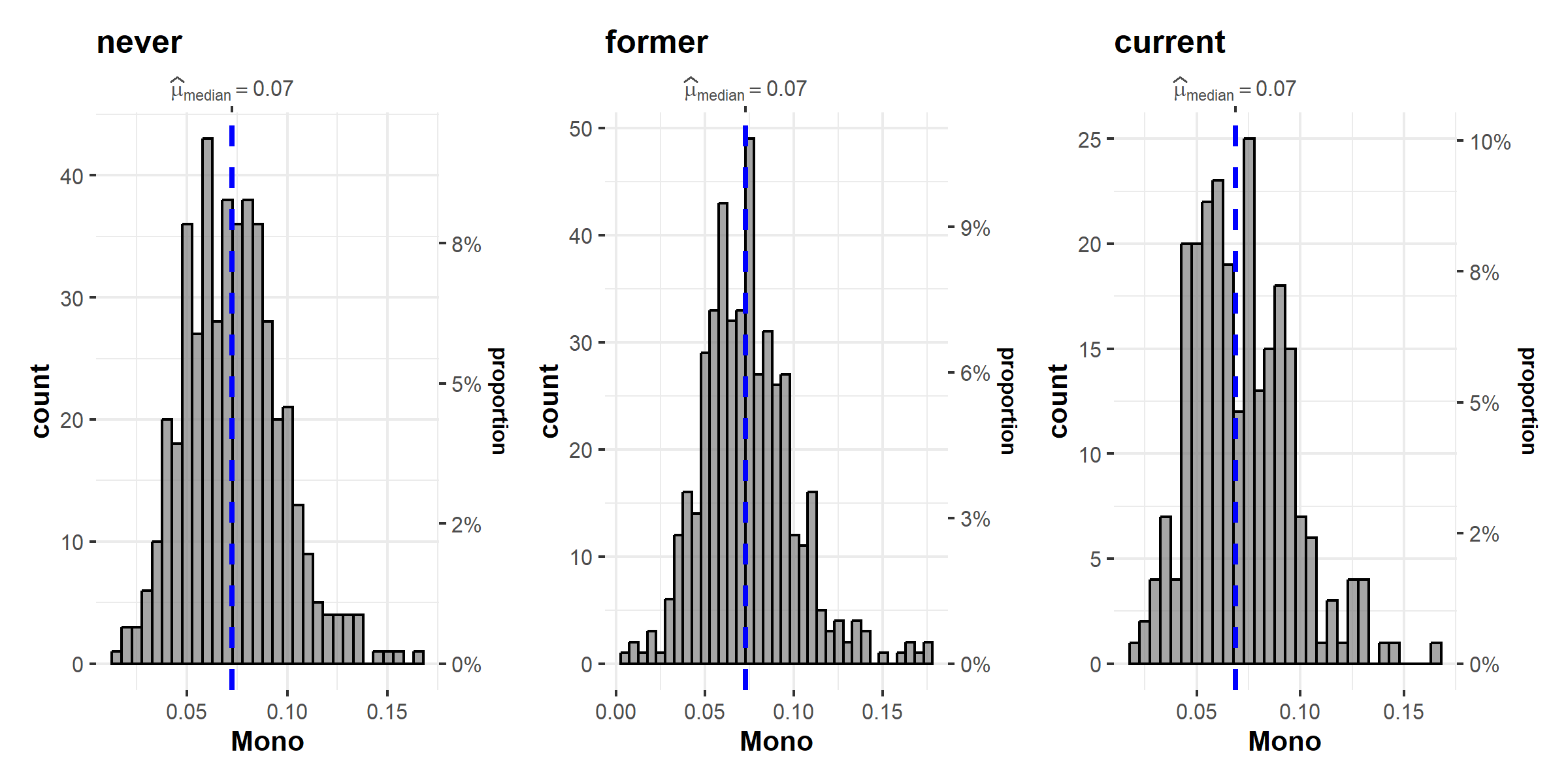


**Supplementary Figure 12:** Distribution of monocytes calculated by the Houseman algorithm and stratified by smoking status in the replication sample


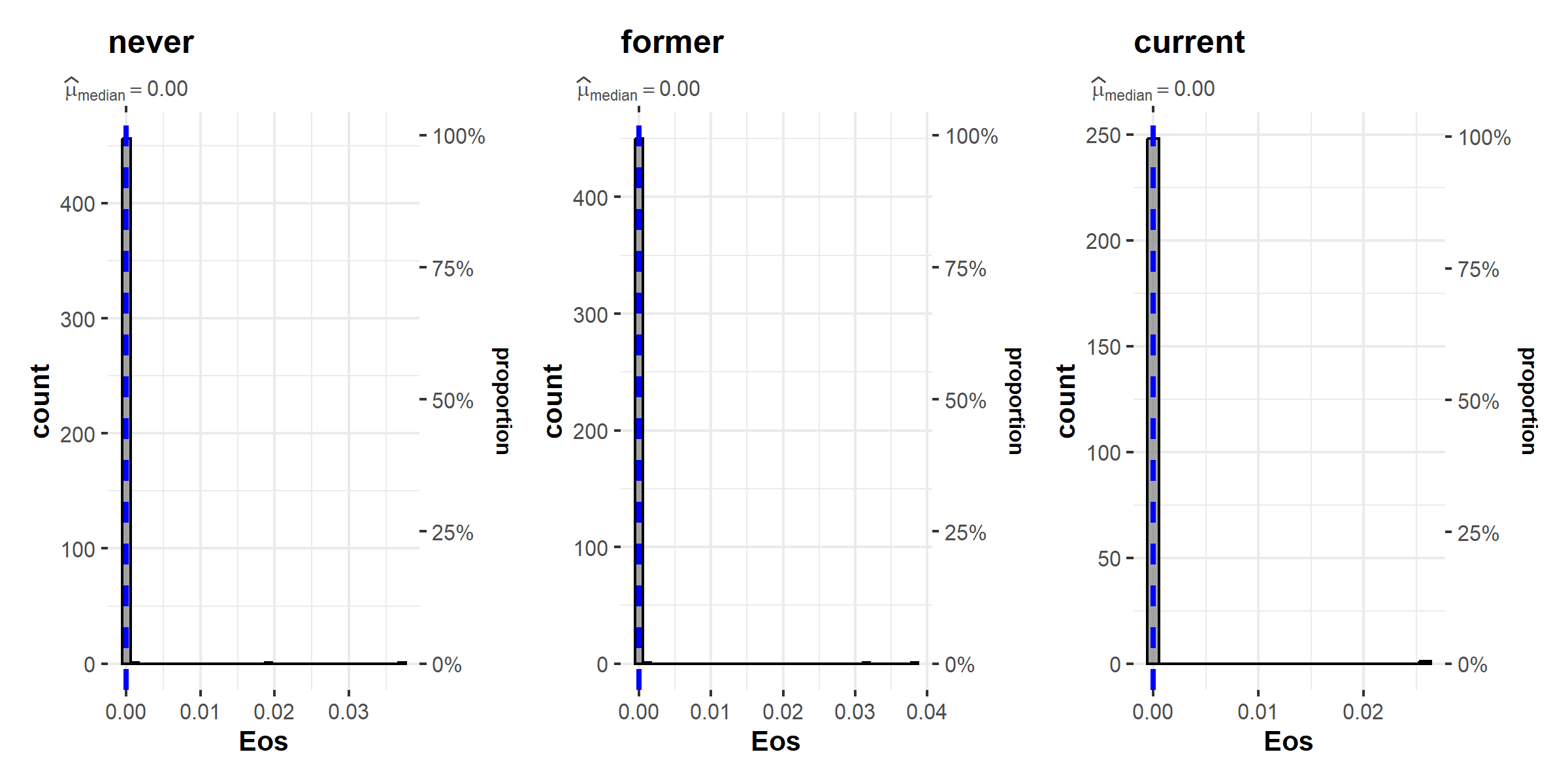


**Supplementary Figure 13:** Distribution of eosinophiles calculated by the Houseman algorithm and stratified by smoking status in the replication sample


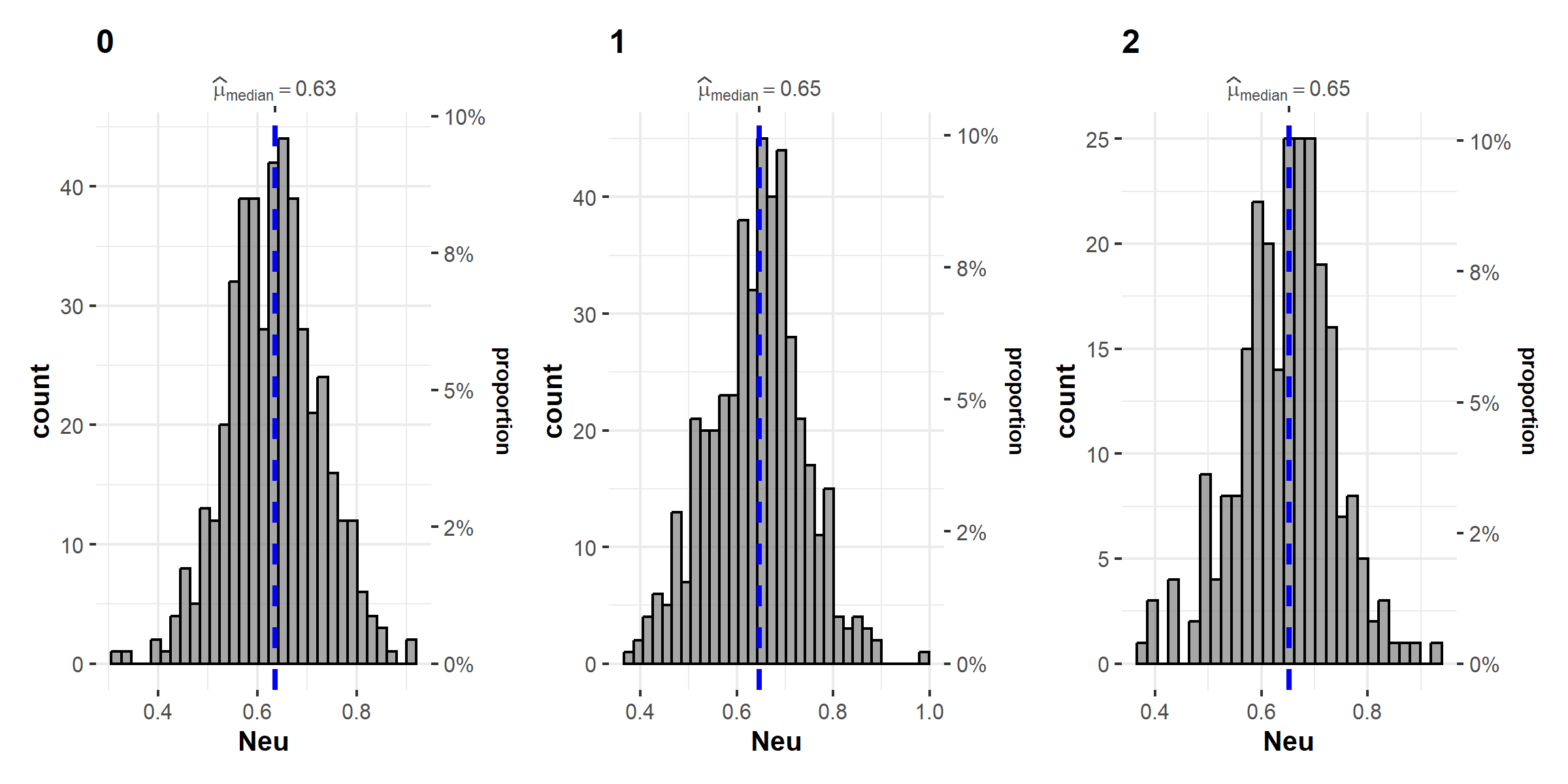


**Supplementary Figure 14:** Distribution of neutrophiles calculated by the Houseman algorithm and stratified by smoking status in the replication sample


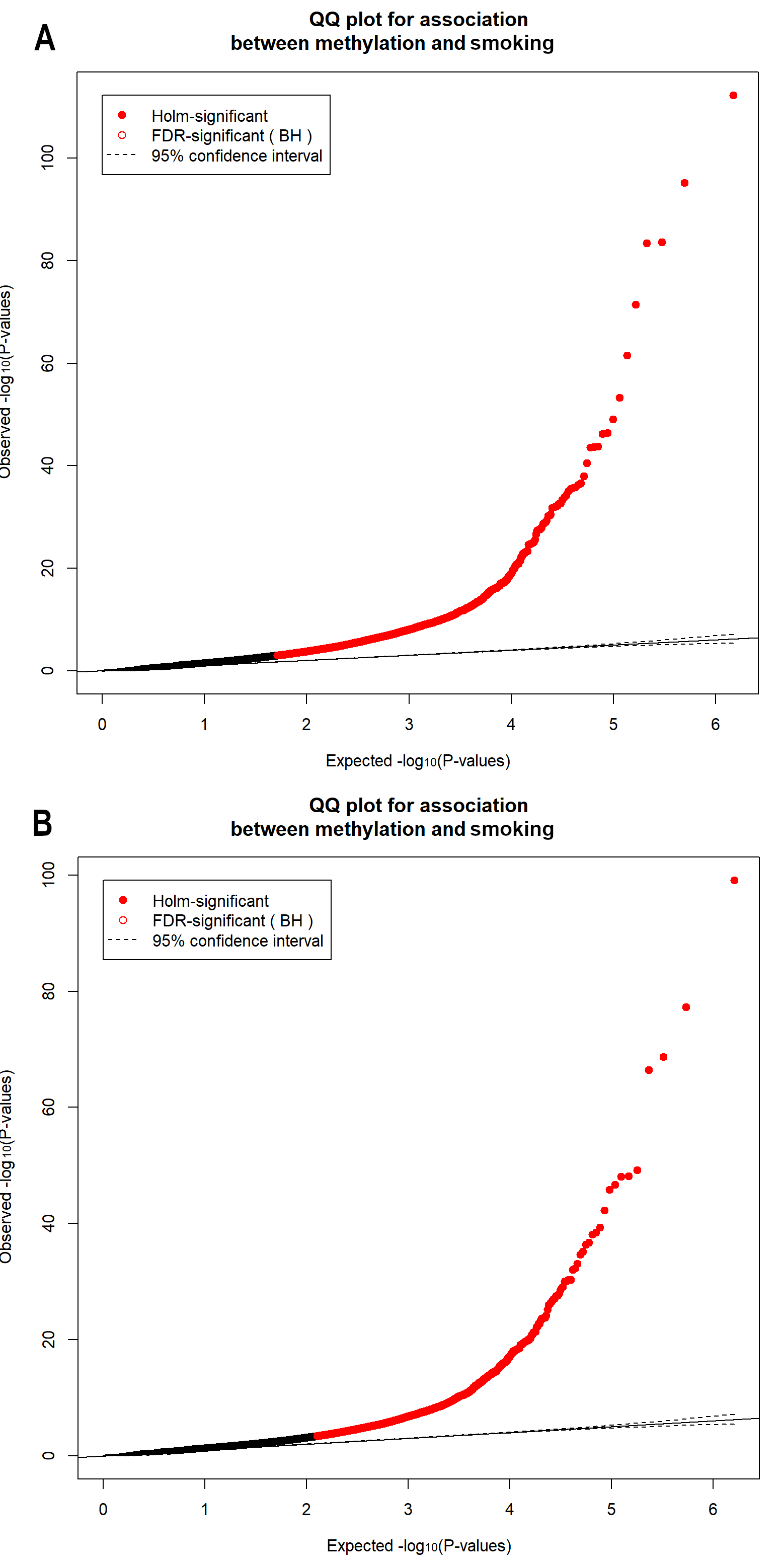


**Supplementary Figure 15:** QQ plots of EWAS of current smokers vs never smokers in the discovery sample(A) and the replication sample (B).


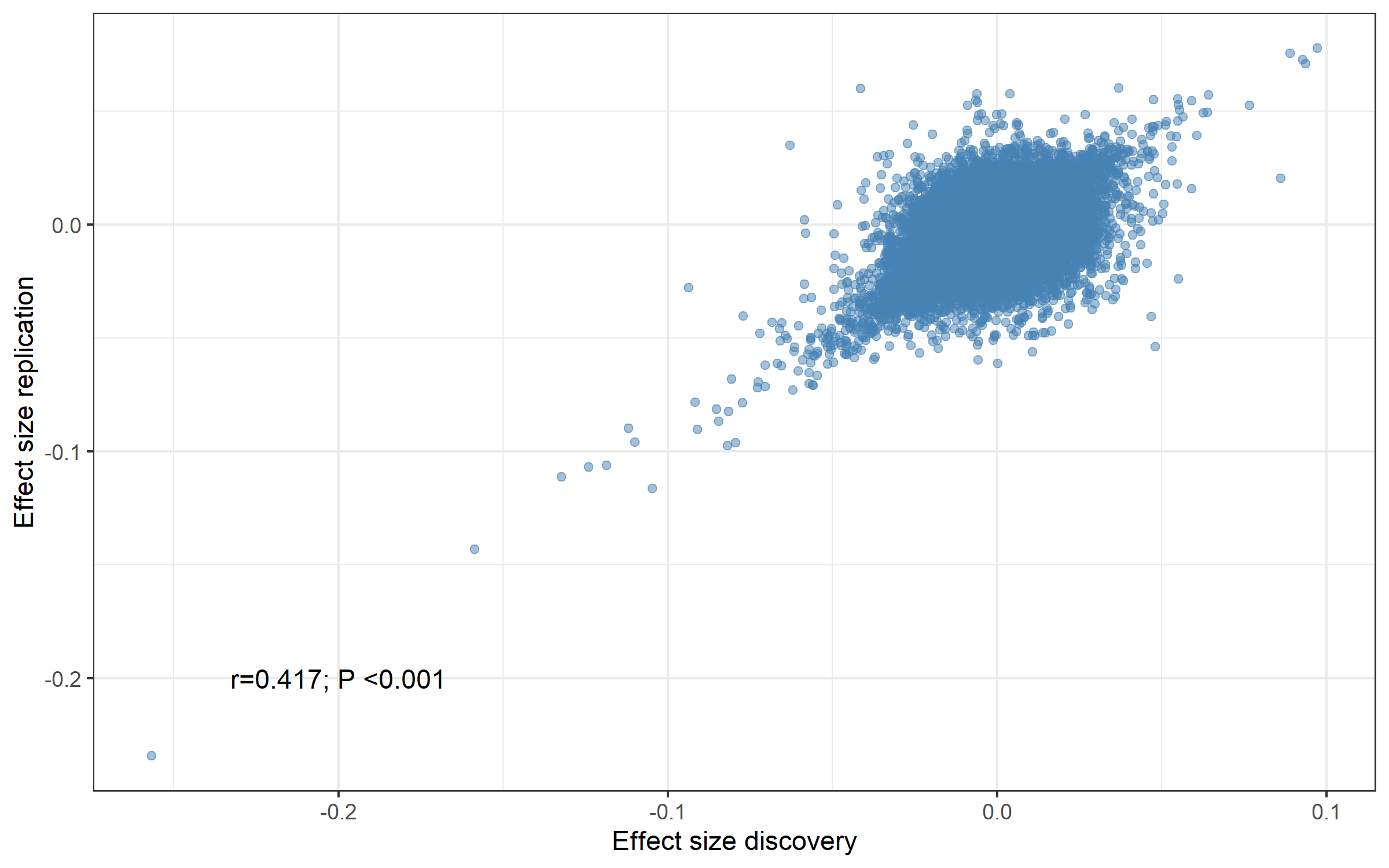


**Supplementary Figure 16:** Scatter plot of the effect sizes in EWAS of current smokers vs never smokers including the Pearson correlation coefficient.


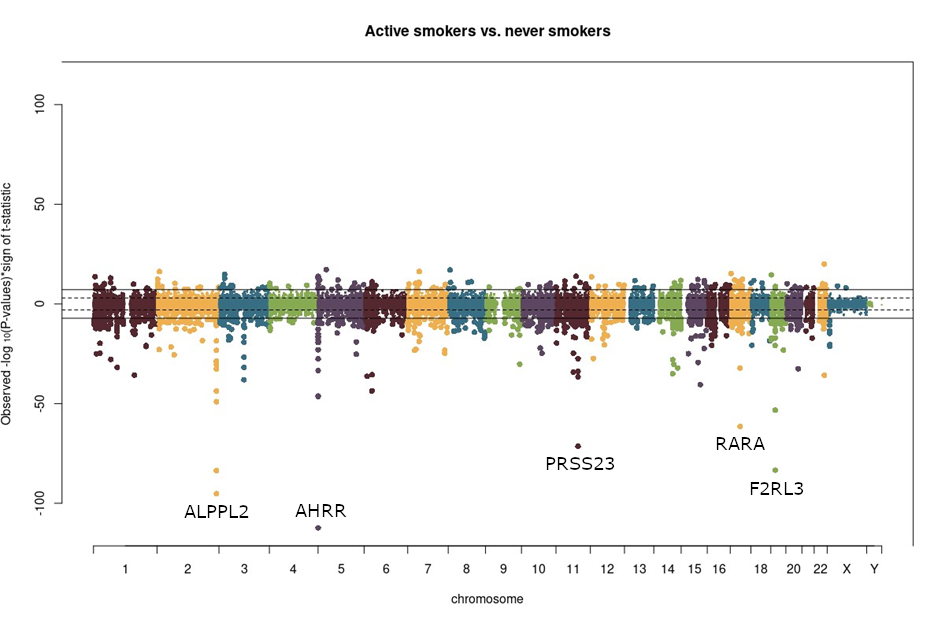


**Supplementary Figure 17**: Reflective Manhattan plot of the analyses of current smoker versus never smoker with smoking status entered as numerical variable. CpGs with hypermethylation depicted in the upper half and CpGs with hypomethylation in the lower half.


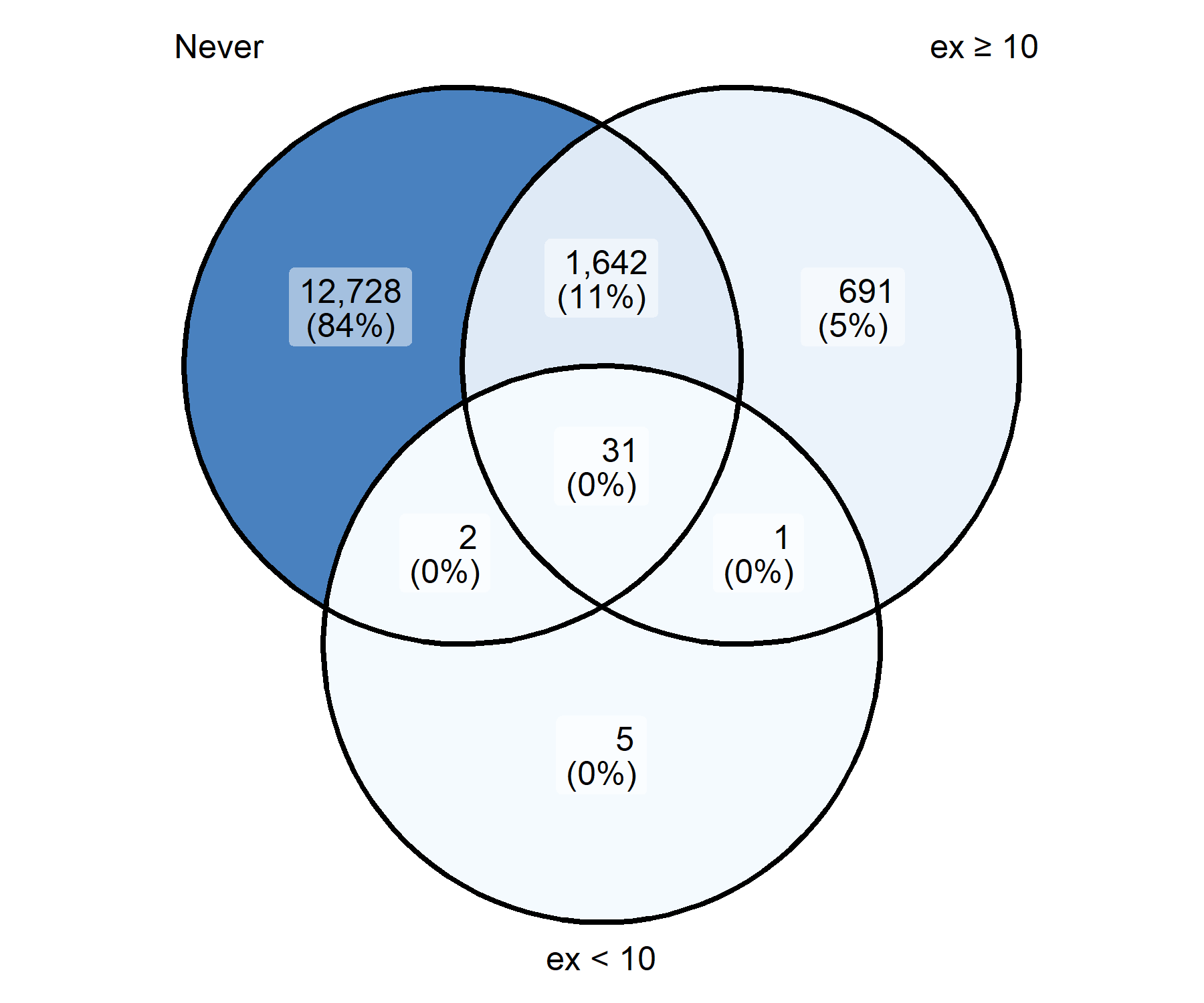


**Supplementary Figure 18:** Venn diagram showing the overlap in the results of the EWAS of current smokers versus never smokers, ex-smokers that quit smoking more than 10 years before study entry and ex-smokers that quit smoking less than 10 years before study entry (number CpGs that were significant after FDR-correction in the analyses).


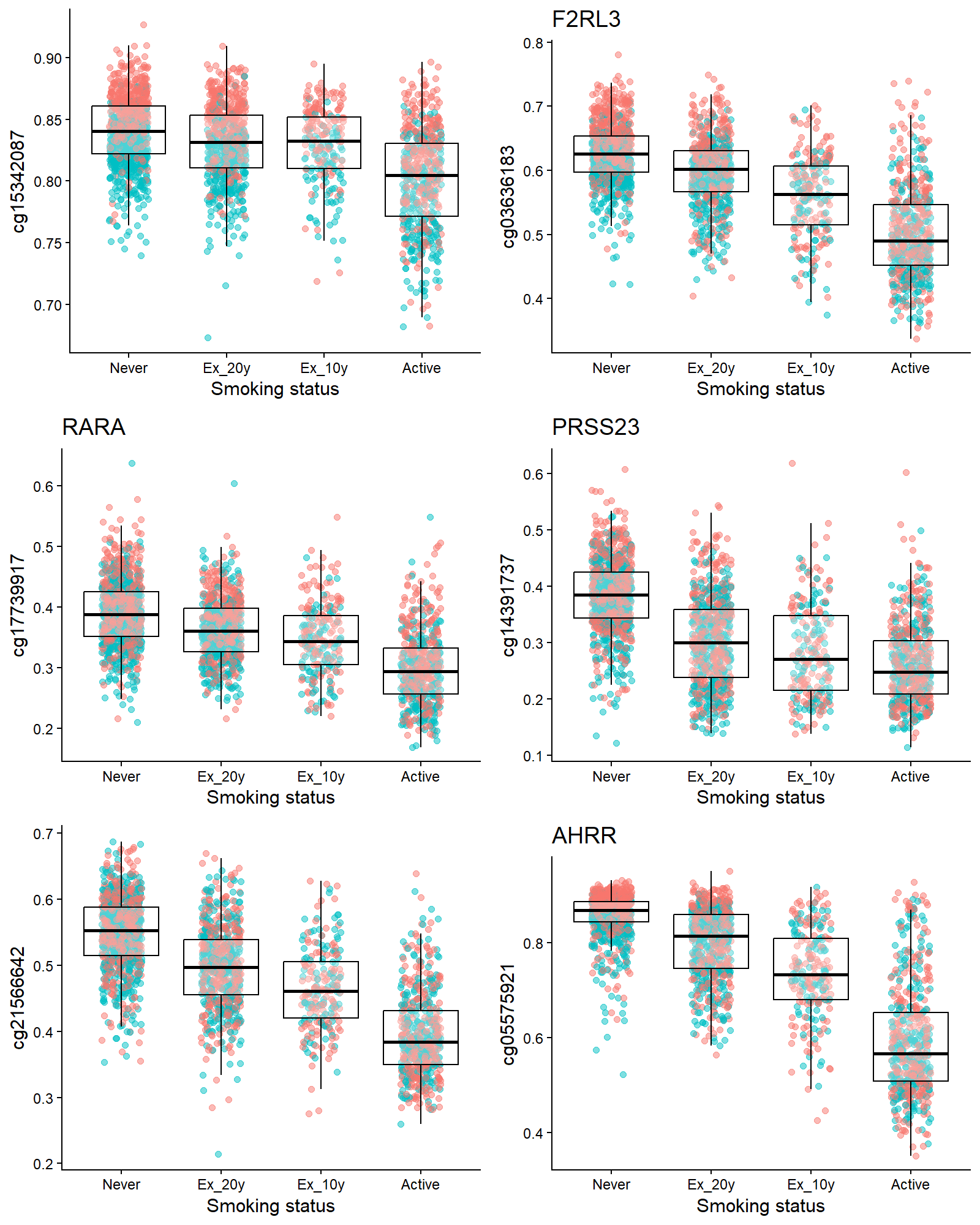


**Supplementary Figure 19:** Distribution of methylation beta values of CpGs at the six most significant loci in the different groups analyzed. Beta values of the individual CpGs on the y-axis and smoking status on the x axis. Individual data points are shown dots (blue: discovery sample, red: replication sample) and overlaid by box plots. The line shows the median value and 50% of all values (the 2nd and 3rd quartile) are included in the box.


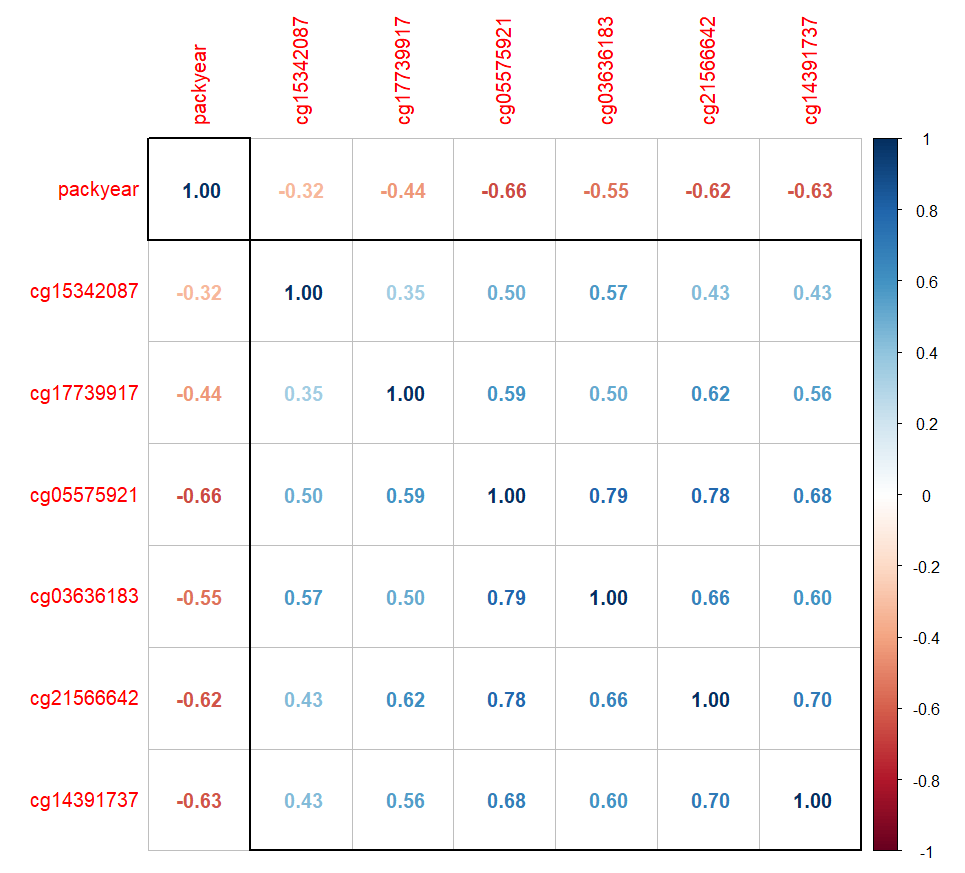


**Supplementary Figure 20:** Correlation of top DMPs with pack-years. Shown are coefficients from Spearman correlation analysis.


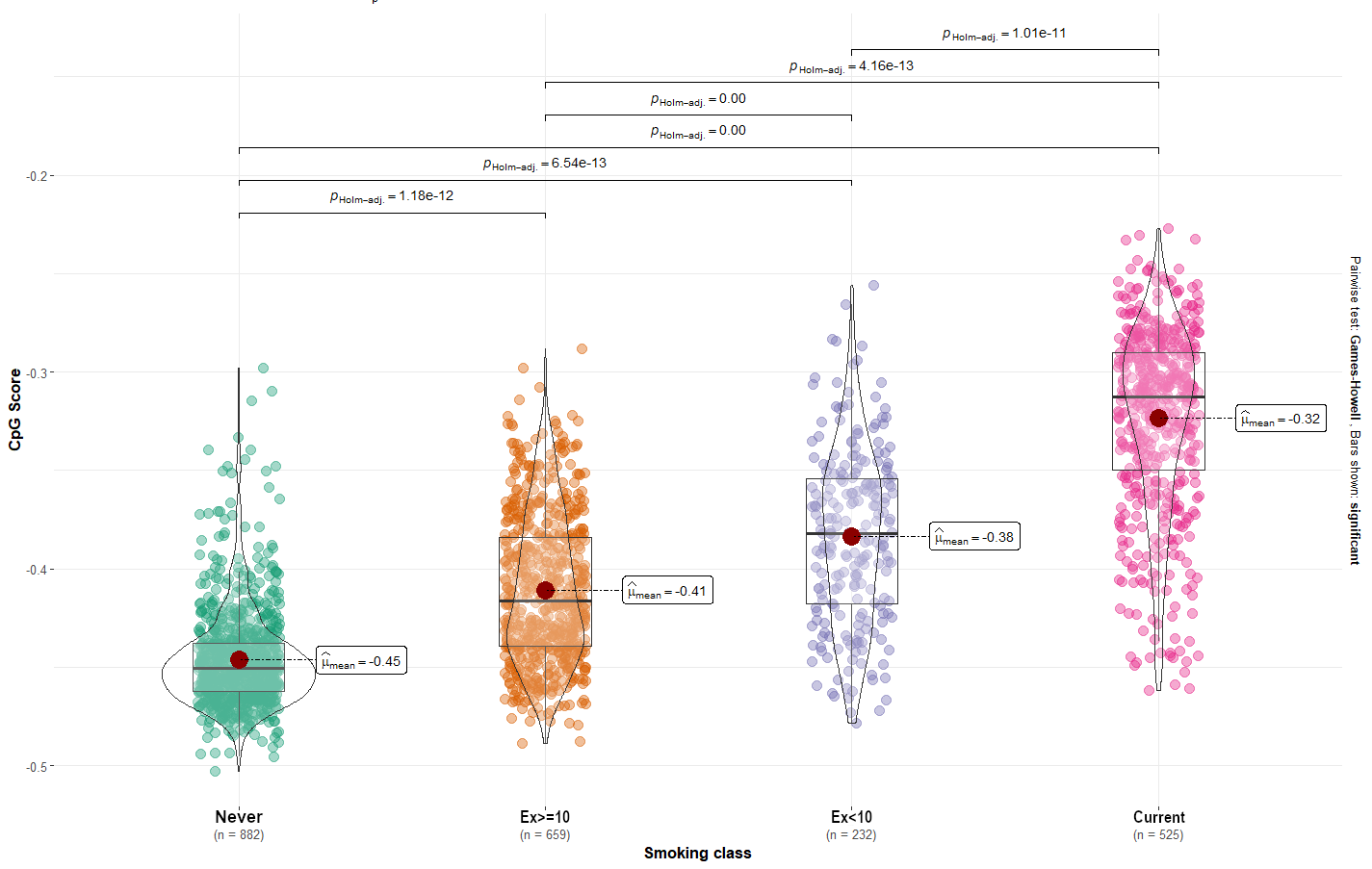


**Supplementary Figure 21**: Distribution of the CpG score in never smokers, former smokers and current smokers.


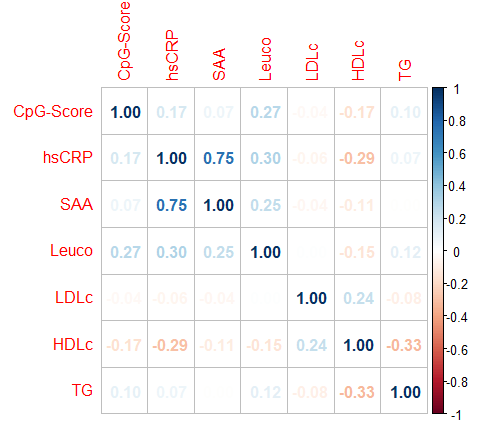


**Supplementary Figure 22**: Spearman correlation between the CpG score and markers of inflammation and lipid metabolism
